# Genetic Dissection of Photoreceptor Subtype Specification by the *Drosophila melanogaster* Zinc Finger Proteins Elbow and No ocelli

**DOI:** 10.1371/journal.pgen.1004210

**Published:** 2014-03-13

**Authors:** Mathias F. Wernet, Kerstin M. Meier, Franziska Baumann-Klausener, Ruslan Dorfman, Ulrich Weihe, Thomas Labhart, Claude Desplan

**Affiliations:** 1 Center for Developmental Genetics, Department of Biology, New York University, New York, New York, United States of America; 2 Department of Neurobiology, Stanford University, Stanford, California, United States of America; 3 Institute of Molecular Life Sciences, University of Zurich, Zurich, Switzerland; 4 Geneyouin Inc., Maple, Ontario, Canada; 5 European Molecular Biology Laboratory, Heidelberg, Germany; Vesalius Research Center, Belgium

## Abstract

The *elbow/no ocelli* (*elb*/*noc*) complex of *Drosophila melanogaster* encodes two paralogs of the evolutionarily conserved NET family of zinc finger proteins. These transcriptional repressors share a conserved domain structure, including a single atypical C2H2 zinc finger. In flies, Elb and Noc are important for the development of legs, eyes and tracheae. Vertebrate NET proteins play an important role in the developing nervous system, and mutations in the homolog ZNF703 human promote luminal breast cancer. However, their interaction with transcriptional regulators is incompletely understood. Here we show that loss of both Elb and Noc causes mis-specification of polarization-sensitive photoreceptors in the ‘dorsal rim area’ (DRA) of the fly retina. This phenotype is identical to the loss of the homeodomain transcription factor Homothorax (Hth)/dMeis. Development of DRA ommatidia and expression of Hth are induced by the Wingless/Wnt pathway. Our data suggest that Elb/Noc genetically interact with Hth, and we identify two conserved domains crucial for this function. Furthermore, we show that Elb/Noc specifically interact with the transcription factor Orthodenticle (Otd)/Otx, a crucial regulator of rhodopsin gene transcription. Interestingly, different Elb/Noc domains are required to antagonize Otd functions in transcriptional activation, versus transcriptional repression. We propose that similar interactions between vertebrate NET proteins and Meis and Otx factors might play a role in development and disease.

## Author Summary

The eyes of many animals contain groups of photoreceptor cells with different chromatic sensitivities that can be arranged in complex patterns. It is of great interest to identify the genes and pathways shaping these ‘retinal mosaics’ which include stochastically distributed groups of cells, versus highly localized ones. In many insect eyes, which are composed of large numbers of unit eyes, or ommatidia, specialized photoreceptors are found only in the dorsal periphery, where they face the sky. These ommatidia are responsible for detecting linearly polarized skylight, which serves as an important navigational cue for these animals. Here we describe how two closely related proteins called Elbow and No ocelli interact with the transcription factors Homothorax and Orthodenticle to correctly specify the polarization detectors at the dorsal rim of the retina of *Drosophila melanogaster*. Interestingly, all four proteins are evolutionarily conserved from worms to humans, and they appear to be involved in similar developmental processes across species. Furthermore, human homologs of Elbow and No ocelli have been identified as promoters of luminal breast cancer. The newly identified role of these two proteins within a regulatory network might therefore enable new approaches in a number of important processes.

## Introduction

The developing retina of *Drosophila melanogaster* is a powerful model for studying the specification of cell fates within a retinal mosaic. One important aspect is the localized specification of photoreceptor cell types in response to Wg/Wnt signaling. Wg emanating from the head cuticle specifies specialized ommatidia at the dorsal rim of the developing retina [1]–[3]. Here we show that the *Drosophila* NET family zinc finger proteins Elbow (Elb) and No ocelli (Noc) play a crucial role in this process. In fly wing imaginal discs, as well as in a mouse breast cancer model, NET proteins inhibit Wingless (Wg)/Wnt signaling [4]–[6], while expression of the *C. elegans* homolog TLP-1 is regulated by Wg/Wnt signaling [7]. Hence, the patterning of the ommatidial mosaic in the dorsal periphery of the retina serves as an attractive model to characterize the different roles of NET proteins.

**Figure 1:**
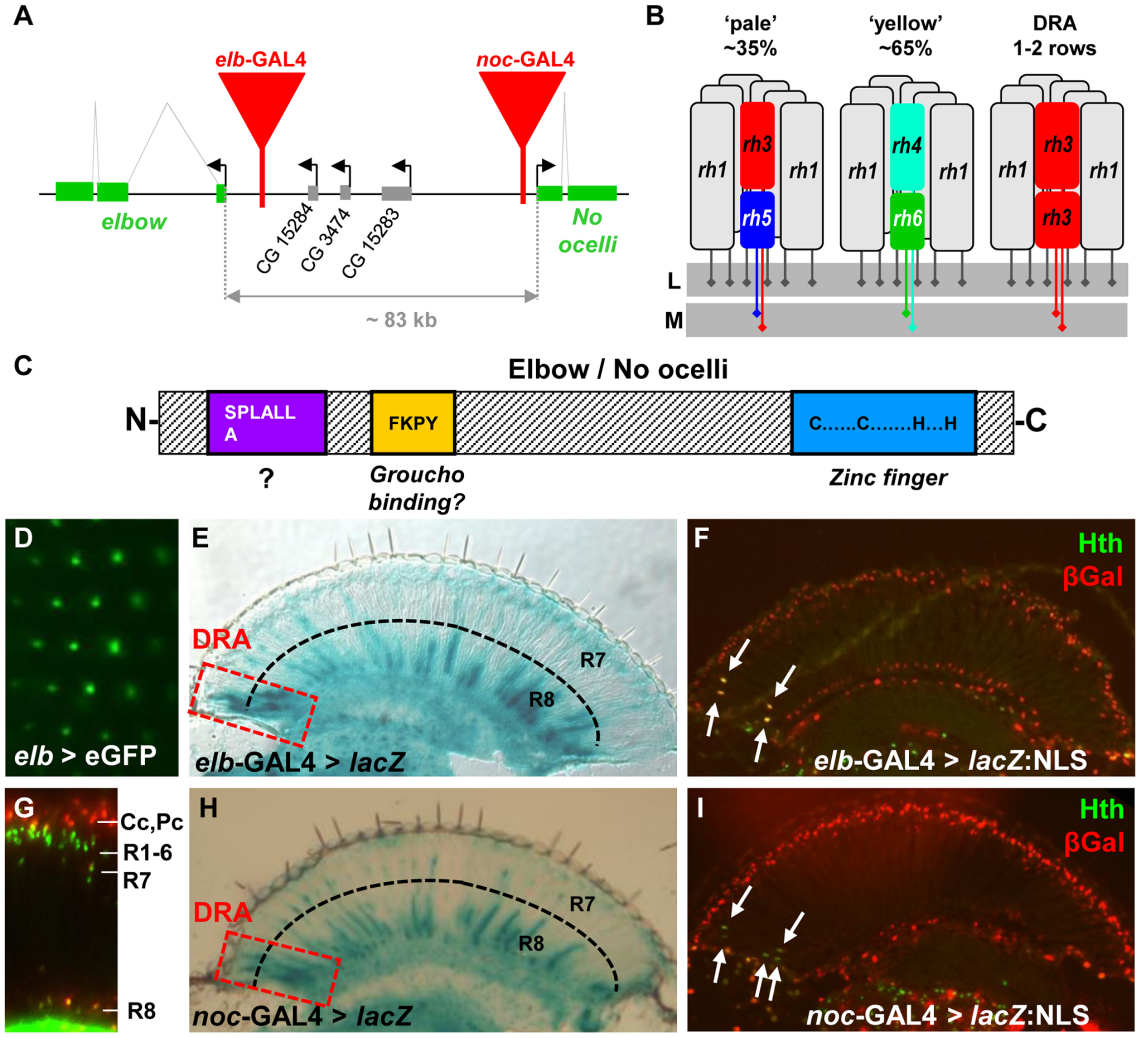
Expression of *elbow* (*elb*) and *No ocelli* (*noc*) in photoreceptors. **A.** Schematic of the *elb*/*noc* locus with GAL4 enhancer traps shown as red triangles. **B.** Schematic summarizing Rhodopsin expression in the three main ommatididal subtypes of *Drosophila*. L  =  lamina; M  =  medulla. **C.** Domain structure of Elb/Noc proteins: like most members of the NET family of transcriptional repressors they contain a single C2H2 zinc finger domain at the C-terminus (light blue), as well as a conserved SPLALLA motif of unknown function at the N-terminus (purple). In between the two lies a Groucho-binding motif (yellow). **D.** GFP fluorescence of *elb* > UAS-eGFP observed under water immersion. Fluorescence localizes to the central photoreceptors of each ommatidium (R7 or R8), as well as non-neuronal cells (green blur). **E.** Cryostat cross section through an adult eye expressing βGalactosidase (*elb* > *lacZ*). Strong expression was observed in R7 and R8 of the Dorsal Rim Area (red dashed box). Additional expression exists in the brain, in many R8 cells, as well as few R7 cells throughout the retina. **F.** Elbow expression (*elb* > *lacZ*:NLS; red) co-localizes with DRA marker Homothorax (Anti-Hth; green; white arrows). **G.** Expression of *elb*-GAL4 driving UAS-*lacZ*:NLS in the adult retina (visualized on a Cryostat cross section): expression is restricted to R7 and R8 nuclei, as well as non-neuronal cone cell nuclei above the R1-6 level. **H.** Adult expression of *noc*-GAL4 is virtually indistinguishable from *elb*. Expression is strongest in R7 and R8 in the DRA (red dashed box), as well as subsets of R7 and R8 cells outside the DRA. **I.** *No ocelli* expression (*noc* > lacZ:NLS; red) also co-localizes with DRA marker Homothorax (Anti-Hth; green; white arrows).

The *elbow/no ocelli* complex of *Drosophila* is a ∼200 kb locus encoding two closely related proteins belonging to the NET family of zinc finger proteins [8]–[10] (Figure 1A). These proteins are related to Sp1-like transcription factors, but contain only one atypical C2H2 zinc finger with 8 amino acids between the two crucial cysteine residues, making these proteins unlikely to bind DNA directly (for review: [11]). They appear to function as repressors of transcription [12], [13] and contain several conserved amino acid motifs (Figure 1C). Elb and Noc bind the co-repressor Groucho through a conserved FKPY motif [10], while other NET family members use different domains for this interaction [6], [14]. The third highly conserved domain of NET proteins is an N-terminal ‘Sp motif’, a SPLALLA amino acid sequence shared with the vertebrate Sp1 family of transcription factors, but not with *Drosophila* Sp1 or Buttonhead (Btd) [7], [11]. Roles for this motif in protein degradation or transcriptional repression have been suggested [15], [16]. In vertebrates, NET family factors play an important role in the specification of motorneurons [17], and in the developing hindbrain [18], [19] and striatum [20], [21]. Loss of one NET family member, ZNF703, in humans promotes luminal breast cancer [22], [23]. In flies, Elb and Noc are important for proximo-distal patterning of the legs and for morphogenesis of tracheal branches [10], [24].

The *Drosophila* compound eye is composed of approximately 800 ommatidia (unit eyes), each containing 8 photoreceptor neurons (called R1 to R8), as well as pigment, cone, and bristle cells [25]. The outer photoreceptors (R1-6) have short axon fibers that terminate in the first optical ganglion, the lamina. The two remaining inner photoreceptors R7 and R8 have light-gathering structures (rhabdomeres) that are located in the center of the ommatidium, R7 situated on top of R8. Their long axonal fibers terminate in a deeper layer of the optic lobe, the medulla [26]. Specification of R7 and R8 depends on the *Spalt* complex (*Sal*), encoding two transcription factors, Spalt major (Salm) and Spalt related (Salr) [27]. The R7 cell type is then induced by Prospero (Pros) [28], while Senseless (Sens) determines R8 [29], [30].

A retinal mosaic arises from the molecular differences between R7/R8 of different ommatidia, creating functional heterogeneity [31] (Figure 1B). At least four ommatidial subtypes can be distinguished in flies. Two subtypes named ‘pale’ (**p**) and ‘yellow’ (**y**) are distributed randomly throughout the eye, with a ratio of 35% (**p**) to 65% (**y**) [32], [33]. R7 cells in **p** ommatidia always express UV-sensitive rhodopsin Rh3, while the underlying R8 express a blue-sensitive rhodopsin Rh5 [34], [35]. In **y** ommatidia, R7 express another UV-sensitive rhodopsin, encoded by the *rh4* gene, while R8 cells contain a green-sensitive Rhodopsin, Rh6 [36]–[38]. This mosaic of chromatic sensitivities created by p/y ommatidia provides the substrate for *Drosophila* color vision [39], [40]. In the dorsal-most third of the adult eye, y ommatidia show an additional specialization by co-expressing both UV Rhodopsins Rh3 and Rh4 in R7 cells [41]. Finally, a narrow band of ommatidia along the dorsal head tissue, called the ‘dorsal rim area’ (DRA), manifests morphological and molecular specializations making these ommatidia ideal detectors for polarized light [42]. Inner photoreceptors R7 and R8 of DRA ommatidia are monochromatic since they both contain the UV Rhodopsin Rh3 [43]. Furthermore, the diameter of their rhabdomeres is enlarged, and the absence of rhabdomeric twist in DRA ommatidia results in high polarization sensitivity [42], [44], [45]. As a consequence, DRA ommatidia are both necessary and sufficient for detecting linearly polarized light emanating from the sky [45], [46].

We have shown that the development of DRA ommatidia in *Drosophila* depends on Homothorax (Hth) a homeodomain transcription factor homologous to vertebrate Meis factors [2],[47]. Hth is both necessary and sufficient for the specification of DRA ommatidia where it is expressed in both R7 and R8 [2]. Hth always co-localizes in nuclei with its ubiquitous cofactor Extradenticle (Exd), whose nuclear localization depends on Hth (for review: [48]). We have shown that the transcription factor Orthodenticle (Otd) is required as an activator of *rh3* expression in DRA ommatidia [49]. Otd is expressed in all adult *Drosophila* photoreceptors, where it acts as a general activator of *rh3* and *rh5* expression [49], [50]. Otd also induces repression of *rh6* through induction of another homeodomain transcription factor, the repressor ‘Defective proventriculus’ (Dve). In outer photoreceptors (R1-6), Otd and Dve act in a feedforward loop, resulting in repression of *rh3 rh5* and *rh6* by Dve in these cells [50]. In inner photoreceptors, Dve is repressed by Spalt (Sal) factors, allowing activation of *rh3* and *rh5* by Otd in ‘pale’ ommatidia. As a consequence, *rh3* and *rh5* are lost in *otd* mutants, while expression of *rh6* is de-repressed into outer photoreceptors, due to the loss of Dve in these cells and the presence of the *rh6-*specific activator Pph13 [51].

Alleles of the gene encoding Otd are named *ocelliless* (*oc*) due to the function of *otd* in patterning the dorsal head cuticle where ocelli form. Therefore, *oc* and *noc* are both required for ocellar development [9], [52]. Furthermore, both genes show specific, overlapping expression at the anterior pole of the fly embryo [53], yet their regulatory relationship is not known. The vertebrate homologs of Otd are involved in retinal development, including OTX1, OTX2 and CRX (‘cone rod homeobox’), whose mutations cause retinal degeneration [54], [55]. Furthermore, OTX1 and OTX2 are required for the specification and regionalization of the forebrain and midbrain, while Otd is required for the development of the anterior brain in flies (for review: [56]). Several aspects of the *otd* mutant phenotype in flies can be rescued by either CRX or OTX2 in the retina [57], as well as the developing brain [58]. Furthermore, many aspects of the OTX1 phenotype in mice can be rescued by substitution with the *Drosophila* Otd protein [59], demonstrating that the molecular function of these factors is conserved.

Here we show that Elb and Noc play an important role in the specification of DRA ommatidia. Loss of both genes together leads to a phenotype identical to the loss of *hth*: the enlarged rhabdomere diameter of DRA inner photoreceptors is lost, and expression of Rh3 in DRA R8 cells is replaced with Rh6. Furthermore, the specific R8 marker Senseless (Sens) becomes de-repressed in R8 cells of DRA ommatidia. Since Hth expression in the DRA is normal in *elb,noc* double mutants, this indicates that Hth is unable to execute its DRA-inducing potential. Gain-of-function experiments in combination with site-directed mutagenesis of the three evolutionarily conserved domains in Elb and Noc reveal that an N-terminal SPLALLA motif as well as the unique zinc finger are crucial for the function of Elb and Noc in DRA ommatidia. Furthermore, Elb and Noc can genetically antagonize the activator or repressor functions of Otd in the retina through distinct protein domains. We therefore propose that NET family proteins might interact with Meis as well as Otx family genes, depending on the transcriptional context.

## Results

### *elbow* and *no ocelli* expression in photoreceptors

In a GAL4 enhancer trap screen for genes expressed in adult photoreceptors, we obtained two independent insertions in the *elbow*/*no ocelli* complex [8], [9], one localized 950 bp upstream of the transcription start of *elbow* (also referred to as ‘*elbow B’, elB,* or *el;* [10], and the other 285 bp upstream of *no ocelli* (*noc;* [24]) (Figure 1A). We previously showed that these two enhancer traps faithfully reproduce the expression patterns of the genes in which they are inserted, in both tracheae and leg imaginal discs [10], [24]. When crossed to UAS-GFP, the GFP signal under water immersion could be localized to the adult inner photoreceptors, with additional signal coming from non-neuronal cells, most likely cone cells (Figure 1D,G). GAL4 expression was strikingly similar between the two lines (Figure 1E,F,H,I). Expression was strongest in R7 and R8 cells in the ‘dorsal rim area’ (DRA), as well as in non-DRA R8 cells (Figure 1E,H), while expression in non-DRA R7 cells was much weaker (Supplemental Figure S1D,E). Different expression levels in R7 and R8 did not correlate with p/y-specific rhodopsin subtypes (Supplemental Figure S1F,G). In DRA ommatidia, strong GAL4 expression always co-localized with Homothorax (Hth) (Figure 1F,I). Although no expression was detected in adult outer photoreceptors R1-6 (Figure 1G), both GAL4 lines were expressed in larval R3 and R4 photoreceptors in eye-imaginal discs, with an onset 3–4 rows posterior to the morphogenetic furrow (Supplemental Figure S1A,B). No expression in R7 and R8 was detected at that stage. Co-expression with the inner photoreceptor marker Spalt (Sal) started around 50% pupation (Supplemental Figure S1C). Thus, *elb*/*noc* expression starts in R3 and R4 early in development, but becomes restricted to inner photoreceptor types during mid-pupal development, and is expressed most strongly in adult R7/R8 of DRA ommatidia, as well as non-DRA R8 cells.

### Loss of typical DRA rhodopsin expression in *elbow*, *no ocelli* double mutants

**Figure 2:**
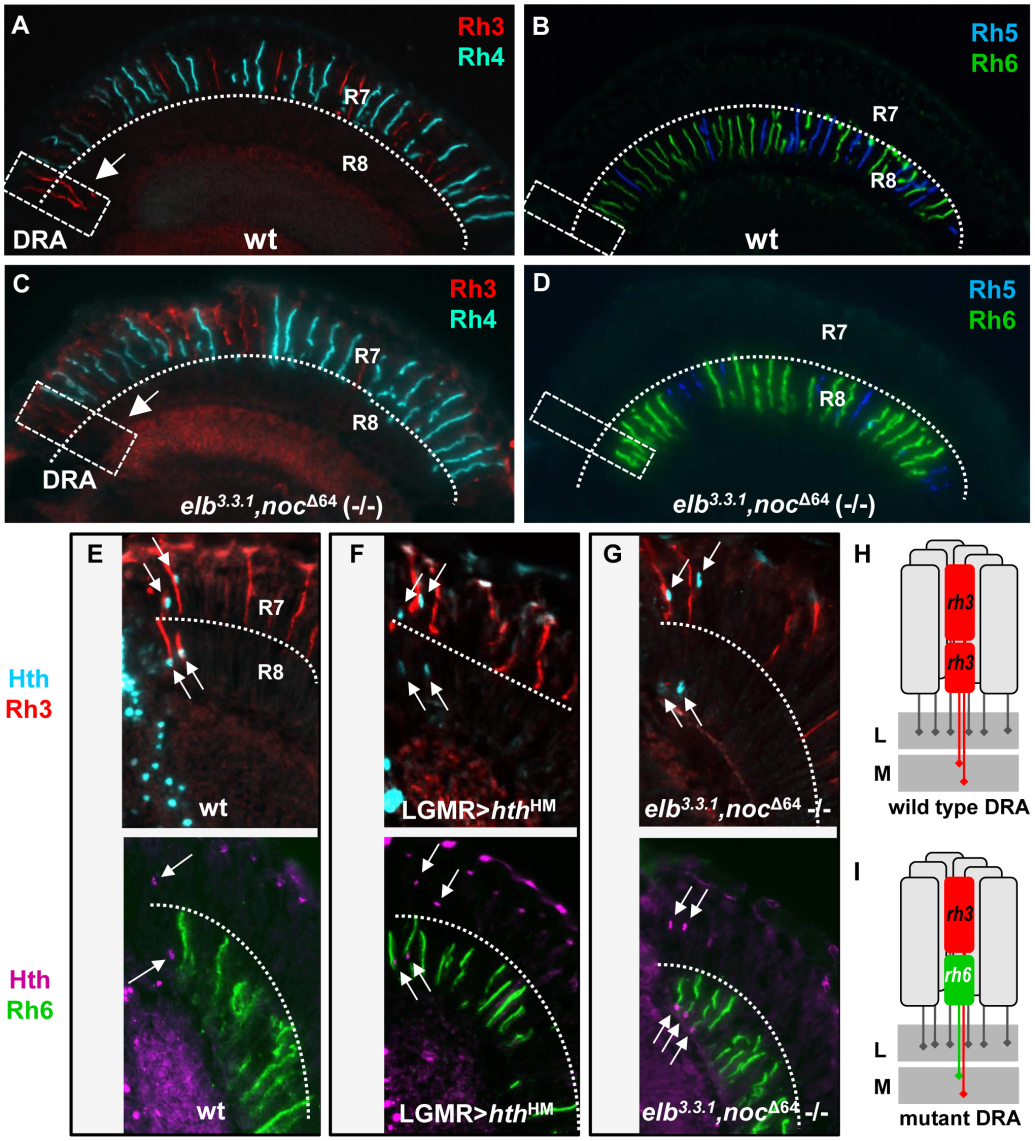
The opsin phenotype of *elb,noc* double mutants is restricted to DRA ommatidia. **A.** Random expression of R7 opsins Rh3 (red) and Rh4 (cyan) in wild type flies. Note expansion of Rh3 into the R8 layer in DRA ommatidia (white dashed box, arrow). **B.** Expression of R8 opsins Rh5 (blue) and Rh6 (green) in wild type flies. Both opsins are excluded from the DRA. **C.** Expression of R7 opsin in *elb,noc* double mutants appears largely normal, but Rh3 expression is lacking in DRA R8 cells (white dashed box, arrow). **D.** In *elb,noc* double mutants, expression of Rh6 (green) appears to extend all the way to the DRA. **E**–**H.** *elb,noc* double mutants phenocopy the opsin phenotype of *homothorax* loss-of-function in DRA. **E.** Top: wild type opsin expression in the DRA, marked with Hth (cyan) and Rh3 (red). Bottom: same genotype marked with Hth (purple) and Rh6 (green). **F.** Upon dominant negative loss of *hth* function (LGMR > *hth^HM^*), Rh6 (green, bottom) expands into DRA R8 cells, whereas R7 cells retain expression of Rh3 (red, top). **G.** Identical phenotype observed in *elb*,*noc* double mutants: co-expression of Rh3/Hth in R7_DRA_ cells (top), and Hth/Rh6 in R8_DRA_ cells (bottom). **H,I.** Summary of DRA phenotypes observed: typical monochromatic Rh3/Rh3 (red) coupling in wild type DRA ommatidia (H) is replaced with ‘odd’ coupled' ommatidia expressing Rh3 in R7 and Rh6 (green) in R8 at the dorsal rim (I), both in *hth* loss-of-function, as well as in *elb,noc* loss-of-function. L  =  lamina; M  =  medulla.

We first tested single mutants for either *elbow* or *no ocelli*, for changes in the Rhodopsin pattern [38], [39]. Neither homozygous viable *elb^3.3.1^* null mutants [24], nor whole mutant eyes generated using the null allele *noc^Δ64^* (-/-) [10], generated with *ey*-Flip/GMR-hid [60], showed a Rhodopsin phenotype affecting R7 cells or R8 cells (Supplemental Figure S2A-F). We then used the same technique to generate elb^3.3.1^,noc *^Δ64^* (-/-) double mutant eyes (Figure 2C,D,G). Although Rhodopsin expression appeared unaffected in most inner photoreceptors (Figure 2A–D, Supplemental Figure S2G,H), Rh3 expression was no longer detected in R8 cells in the DRA (Figure 2C). Instead, the R8 rhodopsin Rh6 was now expressed in the dorsal-most R8 cells (Figure 2D). Mutants lacking Hth function exhibit the same phenotype of ‘odd-coupled’ expression of Rh3/Rh6 in DRA ommatidia R7/R8 [2]. We stained *elb^3^* ^.3.1^, *noc ^Δ64^* (-/-) double mutant eyes for Hth along with Rhodopsins (Figure 2E–G). In these double mutants, Hth expression was unaffected. DRA R7 co-expressed Hth and Rh3 while DRA R8 co-expressed Hth and Rh6 (Figure 2G). This situation was also identical to flies expressing dominant-negative Homothorax (Hth^HM^) in all photoreceptors (Figure 2F). Hence, in the absence of *elb* and *noc*, DRA ommatidia are mis-specified into ‘odd-coupled’ Rh3/Rh6 ommatidia, despite the presence of Hth (Figure 2H). We therefore concluded that Elb and Noc act downstream of, or in parallel to Hth in the specification of DRA photoreceptor cell fates.

### Loss of all DRA markers in *elb*, *no ocelli* double mutants

**Figure 3:**
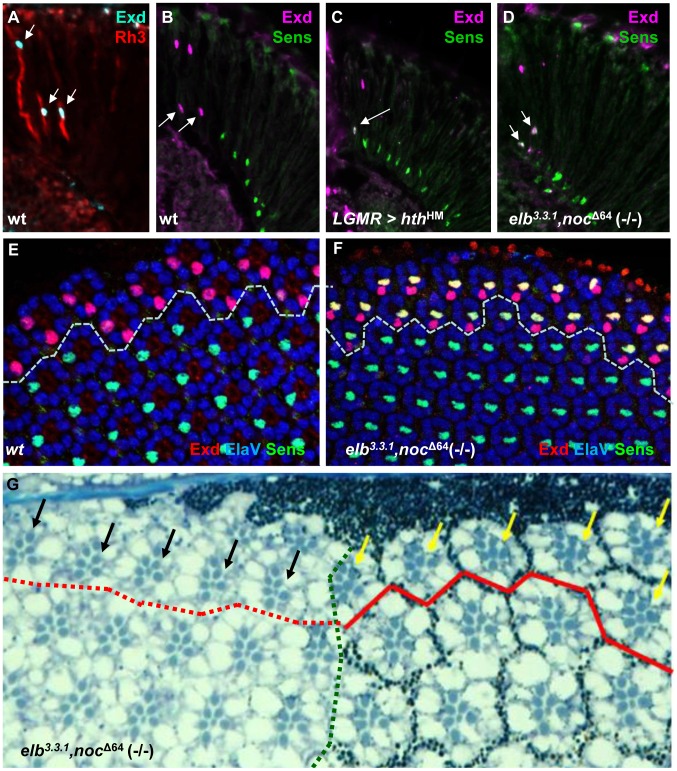
The DRA fate is lost in *elb*,*noc* double mutants. **A.** Nuclear localization of Exd (cyan) in R7 and R8 of wild type DRA ommatidia, marked with Rh3 (red). **B.** Exclusion of R8 marker Senseless (green) from wild type R8_DRA_ cells (arrows), positively marked with Exd (purple). **C**–**F.** Exclusion between Extradenticle (Exd) and Senseless (Sens) is lost in R8_DRA_ cells, in *elb,noc* double mutants (phenocopying *homothorax* loss-of-function). **C.** Co-expression of Exd (indirectly labeled using *hth*-lacZ:NLZ), and Sens (green) in *homothorax* LOF (LGMR > *hth^HM^*). **D.** Identical co-expression of Exd (purple) and Sens (green) is observed in *elb,noc* double mutants. **E.** Exclusion of Exd (red) in the DRA (dashed line) and Sens (green) in a whole mounted pupal retina from wild type flies (labeled with ElaV, blue). Weak co-staining is sometimes observed, at that developmental stage. **F.** Strong co-expression of Exd (red) and Sens (green) in R8 cells of all DRA ommatidia (dashed line), phenocopying *hth* loss-of-function. **G**. Morphological defects of *elb,noc* double mutant DRA ommatidia: typical enlarged rhabdomere diameter of central photoreceptors R7 and R8 (yellow arrows) is lost specifically (black arrows) in *elb,noc* double mutants clones touching the dorsal eye margin (marked by absence of photoreceptor pigment granules to the left of the green dotted line). The same phenotype was described for mutant clones of *hth^B2^* [2]. Note that due to the absence of enlarged inner photoreceptor rhabdomeres the extent of the DRA could not be marked inside *elb*,*noc* mutant tissue (hence the dotted red line).

DRA R8 cells express an R7 Rhodopsin and therefore lack features of R8 cells, like expression of the R8 transcription factor Sens [61]. Indeed, Sens becomes specifically de-repressed in *hth* mutant DRA R8 cells [2]. This phenotype correlates with the gain of Rh6 expression in *hth* mutant DRA R8 cells, since Sens has an inductive effect on *rh6* expression [29], [62]. We stained *elb^3.3.1^,noc ^Δ64^* (-/-) double mutant retinas for Exd and Sens (Figure 3A–F). Like Hth, Exd was localized to the nuclei of DRA R7 and R8 in wild type (Figure 3A,B), as well as in *elb*,*noc* double mutants (Figure 3D; Supplemental Figure S3A,C). However, Sens was now co-expressed with Exd in R8 cells, similar to flies over-expressing dominant-negative Hth^HM^ (Figure 3C). Co-expression of Exd and Sens in DRA R8 cells was already visible in pupal retinas (50% APF; Figure 3E,F), arguing that R8 cells in DRA ommatidia lacking *elb* and *noc* became mis-specified before Rhodopsin expression begins. Outside the DRA, expression of inner photoreceptor markers Spalt, Prospero, and Senseless were indistinguishable from wild type (Supplemental Figure S3B,D–F). Unlike the R8 marker Sens, the R7 marker Prospero is not repressed in DRA R7 cells by Hth/Exd [2]. Hence, there was no immuno-histochemical way to tell whether DRA R7 cells had actually changed their fate in *elb,noc* (-/-) mutants. We therefore assessed the morphology of the eye tissue in tangential plastic sections in *elb* ^3.3.1^,*noc ^Δ64^* (-/-) double mutant eyes (Figure 3G). Wild type DRA ommatidia display an enlarged R7 and R8 rhabdomere diameter [2], [42], [45], [63]. In mutant clones touching the dorsal eye rim, the typical DRA morphology was lost and resembled ommatidia from the main region of the eye. We have previously described the same morphological DRA phenotype in Hth^B2^ (-/-) mutant clones [2]. We therefore concluded that all markers of DRA ommatidia were lost in *elb* ^3.3.1^,*noc ^Δ64^* double mutants, despite persisting expression of Hth.

### Homothorax requires Elb and Noc for inducing DRA fates

**Figure 4:**
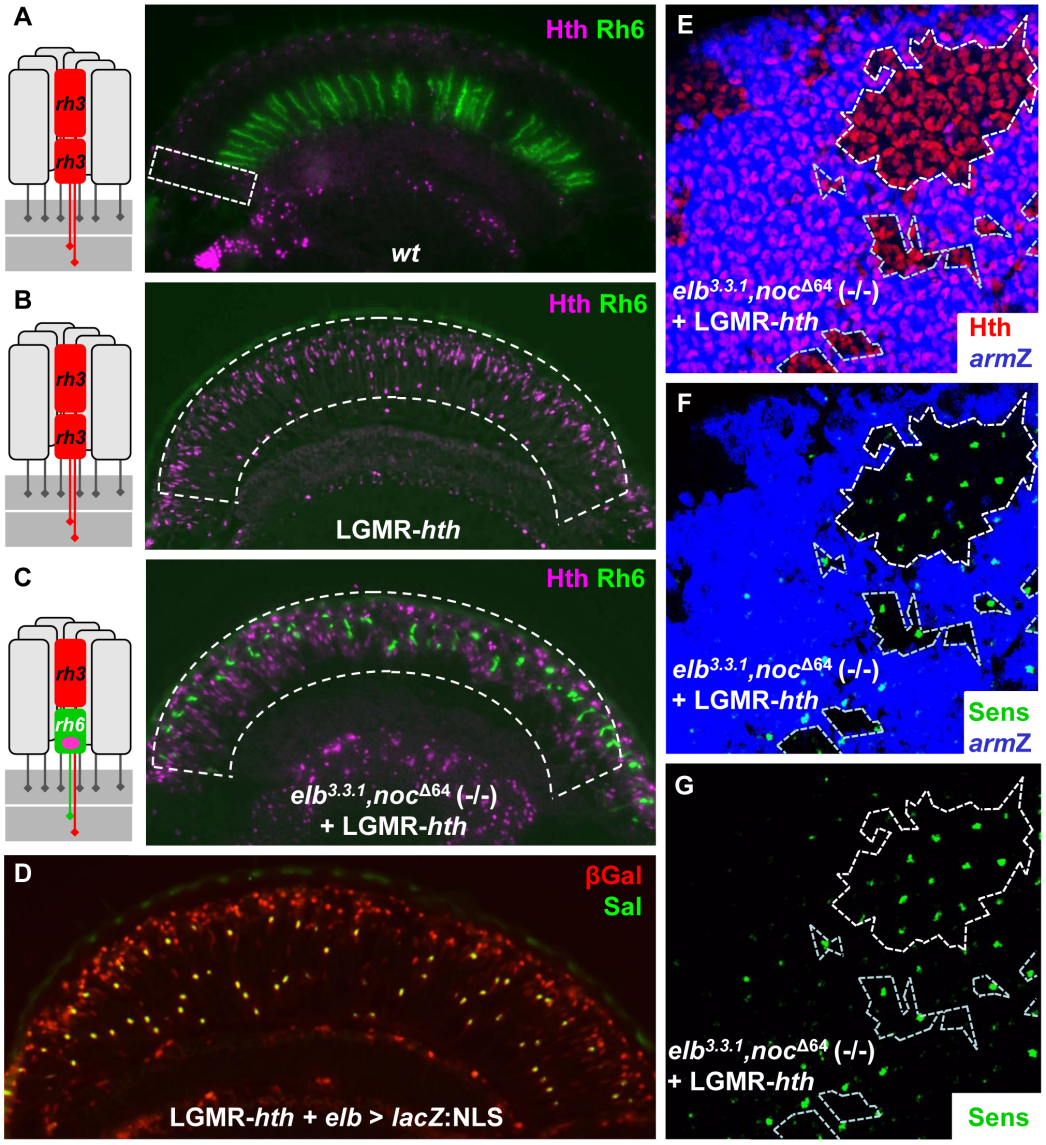
Elb and Noc are crucial for Homothorax function in DRA specification. **A.**–**C.** Homothorax fails to ectopically induce the DRA fate in absence of Elb and Noc. (Ommatidial schematic to the left summarizes the only ommatidial subtype found inside the dashed white boxes; pink dot: nuclear Sens expression in R8). **A.** Exclusion of Rh6 (green) from the DRA (labeled with Hth, purple), in wild type flies (dashed white box). **B.** Complete loss of Rh6 upon over-expression of Hth (purple), under LGMR-GAL4 control. **C.** Co-expression of Rh6 (green) and Hth (purple) in *elb,noc* double mutant flies ectopically expressing Hth (green) in all photoreceptors. **D.** Expression of *elb*-GAL4 (visualized using UAS-lacZ:NLS) is never expanded into R1-6 by over-expression of Hth (green). Instead, strong expression of βGal (red) is observed in all inner photoreceptors R7 and R8 (co-labeled with Spalt, green). **E**–**G.** Homothorax fails to repress Senseless in absence of *elb* and *noc*. Whole mounted pupal retina expressing Hth in all photoreceptors (LGMR-*hth*; red) with homozygous clones lacking both *elb* and *noc* marked by absence of *arm*-*lacZ* (blue, E). The vast majority of strong Sens expression (green) is observed in R8 cells inside homozygous clones (F), as well as in close vicinity to mutant clones (G).

Over-expression of Hth in all photoreceptors leads to a transformation of the entire retina into DRA ommatidia, with Rh3 expression expanding into all inner photoreceptors, while expression of Rh4, Rh5, Rh6, and Sens is lost [2]. We tested whether Elb and Noc were required for this function of Hth, focusing on expression of Rh6 and Sens as the most reliable markers (Figure 4A–C). First, we confirmed that expression of Rh6 was always lost in transgenic flies expressing a GFP:*hth* fusion protein directly attached to LGMR (Figure 4B; Supplemental Figure S4A–C; see Materials and Methods). However, when GFP:*hth* was over-expressed in *elb* ^3.3.1^,*noc ^Δ64^* (-/-) double mutant eyes, Rh6 was found in R8 in the entire retina (Figure 4C). Rh3 was also detected, while Rh4 and Rh5 were absent (data not shown). Thus, the entire retina was transformed into ‘odd coupled’ Rh3/Rh6 ommatidia, like the ones we had described in the DRA of Hth^HM^ mutants. Interestingly, Elb and Noc remained restricted to inner photoreceptors when GFP:*hth* was over-expressed, but became expressed at high levels in all R7 and R8 (Figure 4D). Taken together, these data suggested that Elb and Noc act downstream of Hth in the specification of DRA ommatidia.

As a second test of this epistatic relationship, we took advantage of the fact that the Wg pathway induces DRA ommatidia [1], [3]. Ectopic activation of the Wg pathway with constitutively active forms of Armadillo (*arm* ^S10^, or arm*; [64]) induces Hth in the entire dorsal eye and transforms it into DRA ommatidia, repressing Rh6 in all dorsal R8 cells [2). We therefore tested the requirement of *elb* and *noc* in the dorsal eye using direct GMR-arm* fusions [65]. While Hth was ectopically expressed in all inner photoreceptors of the dorsal eye in GMR-arm* flies, leading to the loss of Rh6 expression from the expanded DRA (Supplemental Figure S4D), we observed co-expression of Rh6 and Hth in R8 throughout the dorsal eye in an *elb* ^3.3.1^,*noc ^Δ64^* (-/-) mutant background containing GMR-arm* (Supplemental Figure S4E). Rh4 and Rh5 were always excluded from the dorsal eye, while Rh3 expression remained (not shown). Hence, activating the Wg pathway in *elb* ^3.3.1^,*noc ^Δ64^* (-/-) mutants induced Hth expression throughout the dorsal half of the eye, transforming it into ‘odd coupled’ Rh3/Rh6 ommatidia. Elb and Noc are therefore necessary for the ability of Hth to induce DRA fate in response to activating the Wingless pathway.

We also tested whether Hth required Elb and Noc for repression of Sens, when ectopically expressed by generating clones of *elb* ^3.3.1^,*noc ^Δ64^* (-/-) mutant tissue in pupal retinas expressing LGMR- *hth* (Figure 4E). In these retinas, the vast majority of R8 cells (though not all) strongly expressing Sens were located within the *elb* ^3.3.1^,*noc ^Δ64^* (-/-) double mutant tissue (Figure 4E–G). Therefore, Hth had lost the ability to repress Sens in the absence of Elb and Noc, which is consistent with a loss of DRA fate [2]. This requirement of Elb/Noc appeared not to be strictly cell autonomous since Sens-positive R8 cells could be observed outside *elb,noc* mutant clones, although almost always in their direct vicinity (Figure 4G) Taken together, we concluded that Hth function in the specification of DRA ommatidia in response Wg pathway activation was dependent on the presence of Elb and Noc.

### Mutations in the Sp/SPLALLA motif and zinc finger affect DRA development

Homothorax is sufficient to induce the DRA fate in all ommatidia when over-expressed [2]. In contrast, over-expression of either Elb or Noc, or both proteins together never resulted in the induction of DRA markers (Supplemental Figure S5), and the gain-of-function phenotypes will be discussed in more detail below. All NET family zinc finger proteins share several conserved protein domains whose functional significance remains incompletely understood [11], [14]. We investigated the role of three domains in DRA specification: the conserved Sp/SPLALLA motif at the N-terminus, a more centrally located FKPY motif that was shown to interact with the transcriptional repressor Groucho, and the unusual zinc finger, located C-terminally (Figure 1C). We altered the amino acid sequence of each of these sequences individually using site-directed mutagenesis and placed the resulting cDNA for *elb* and *noc* in UAS-vectors. Rescue experiments using these point-mutated versions of *elb* or *noc* were not possible since the necessary driver lines *elb*-GAL4 and *noc*-GAL4 were (non-mutant) enhancer traps inserted within the *elb.noc* locus. We therefore tested whether these constructs affected DRA specification in a dominant-negative fashion when over-expressed, similar to Hth^HM^.

**Figure 5:**
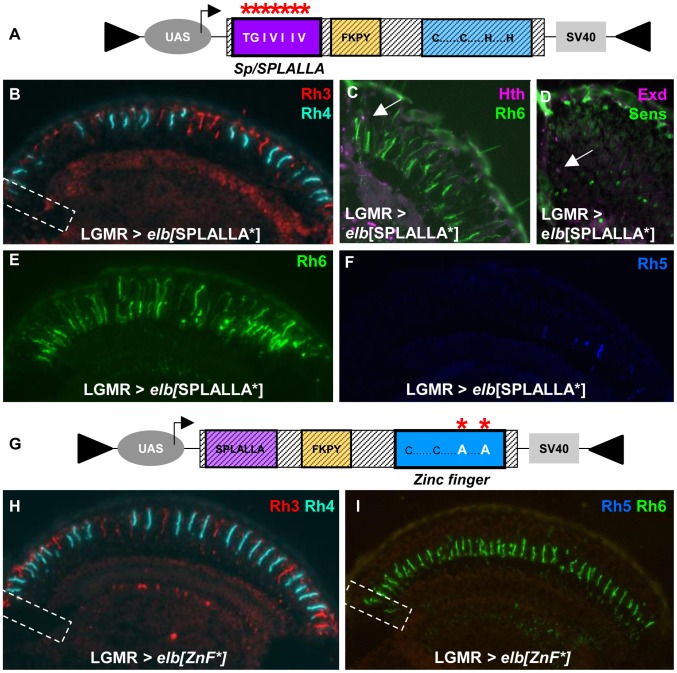
Mutagenesis of Sp/SPLALLA motif or zinc finger transform Elb into a dominant-negative. **A.** Schematic illustrating the UAS-construct for over-expression of an Elb protein with an altered Sp/SPLALLA motif (mutated to TGIVIIV, using site-directed mutagenesis; see Materials and Methods). **B.** Ectopic expression of mutated *elb*[SPLALLA*] using LGMR-GAL4 leads to a loss of typical Rh3 expression in R8_DRA_ cells (white dashed box), while R7 expression appears normal in the rest of the retina. **C.** As a result, Rh6 (green) and Hth (purple) are co-expressed (white arrow) at the dorsal rim of the retina. **D.** Weak co-expression of Sens (green) and Exd (purple) was sometimes observed (white arrow), although not with 100% penetrance. **E.** Expression of Rh6 (green) is strongly expanded into outer photoreceptors R1-6, in LGMR > *elb*[SPLALLA*] flies. **F.** Expression of Rh5 (blue) appears normal in LGMR > *elb*[SPLALLA*] flies. **G.** Schematic illustrating the UAS-construct for over-expression of an Elb protein with a mutated zinc finger (both His were altered to Ala, using site-directed mutagenesis; see Materials and Methods). **H.** Over-expression of mutated *elb*[ZnF*] in all photoreceptors leads to a loss of DRA-specific Rh3 (red) expression in R8_DRA_ cells (white dashed box). **I.** In the R8 cell layer, expression of Rh5 (blue) is specifically lost, and Rh6 (green) is the only remaining R8 rhodopsin, including the DRA (white dashed box).

We altered the Sp/SPLALLA motif of both Elb and Noc to TGIVIIV (Figure 5A; see Materials and Methods). Over-expression of UAS-*elb*[SPLALLA*] in all photoreceptors using LGMR-GAL4 indeed had a dominant negative effect on DRA development. Expression of Rh3 in DRA R8 cells was lost (Figure 5B) and, instead, Rh6 was expanded to the dorsal rim of the retina (Figure 5C). In these DRA R8 cells, we detected co-expression of Sens and Exd, which is an indication of DRA fate loss (Figure 5D). Expression of Rh3, Rh4 and Rh5 was normal outside the DRA in LGMR>*elb*[SPLALLA*] flies (Figure 5F). Interestingly, Rh6 was expanded into all outer photoreceptors R1-6, for reasons we will discuss below.

To test the involvement of the unique zinc finger motif for DRA specification, we mutated the two crucial Histidine residues in the zinc finger of both Elb and Noc proteins, replacing them with Alanine (Figure 5G; see Materials and Methods), thereby disrupting its ability to chelate zinc [66], [67]. Over-expression of *elb*[ZnF*] also resulted in a dominant-negative loss of the DRA: Rh3 was lost in R8 DRA cells and was replaced by Rh6, resulting in Rh3/Rh6 ‘odd-coupled’ ommatidia (Figure 5H,I).

**Figure 6:**
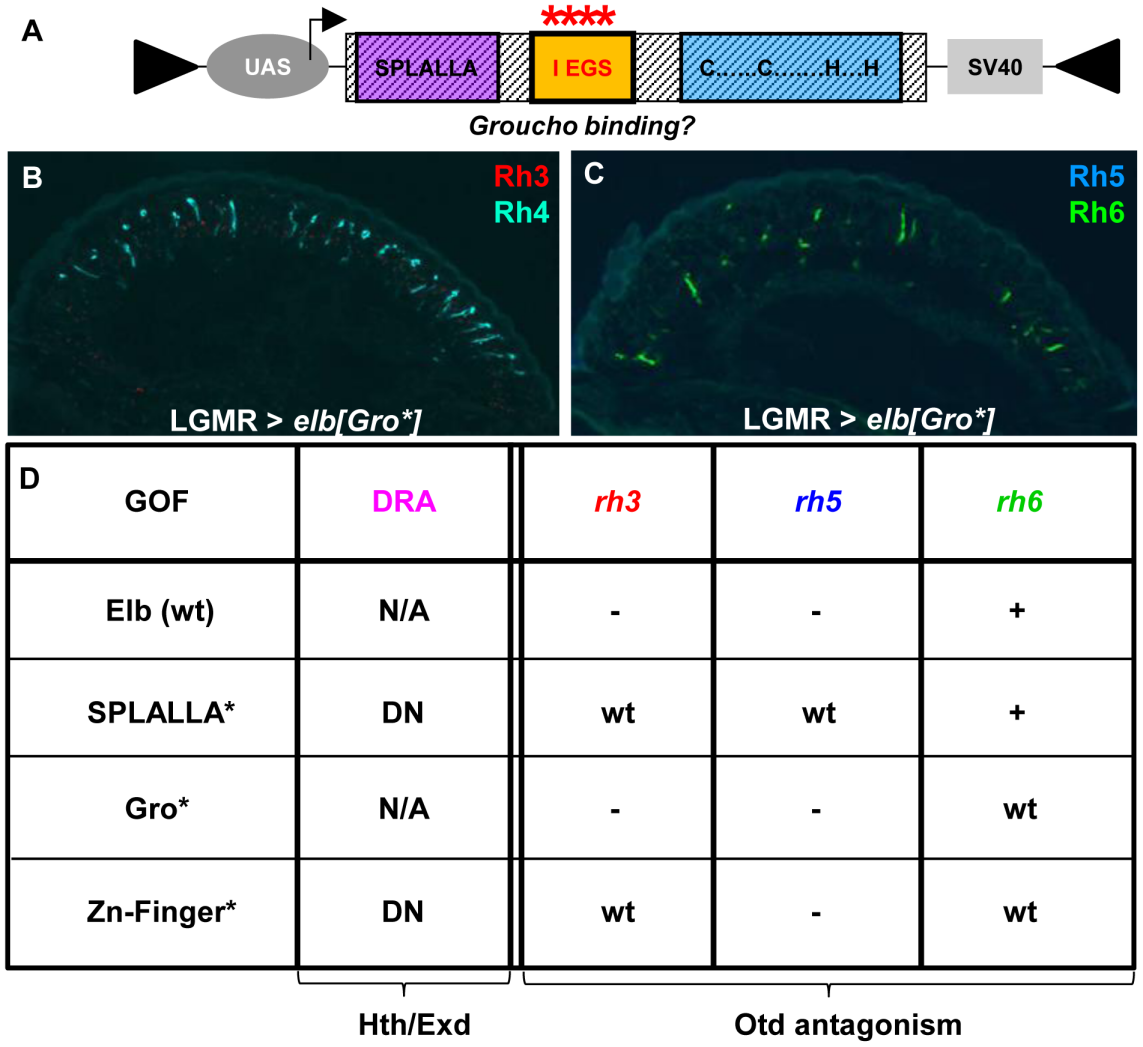
Mutagenesis of conserved Elb domains interferes with different Otd functions. **A.** Schematic illustrating the UAS-construct for over-expression of an Elb protein with an altered Groucho-binding motif (FKPY → IEGS, see Materials and Methods). **B.** Over-expression of mutated *elb*[Gro*] in all photoreceptors using LGMR-GAL4 leads to a specific loss of Rh3 (red), while Rh4 expression (cyan) is normal. **C.** In R8 cells, Rh5 (blue) is specifically lost, while Rh6 expression (green) is normal. **D.** Table summarizing gain-of-function phenotypes observed using different point-mutated forms of Elb. Every mutant produces a specific phenotype that can be broken down into four aspects: DRA specification, *rh3* expression, r*h5* expression, and *rh6* expression. Abbreviations: DN, dominant-negative; wt  =  expression like wild type; ‘-’  =  loss of expression; ‘+’  =  de-repression of expression into outer photoreceptors R1-6; ‘N/A’  =  DRA specification could not be assessed due to ectopic loss of Rh3 expression.

Finally, we altered the conserved, generic Groucho-binding motif present in both Elb and Noc (Figure 6A; FKPY → IEGS; see Materials and Methods). Over-expression of *elb*[Gro*] had no dominant-negative effect on DRA development (Figure 6B,C), and instead resulted in an unrelated rhodopsin phenotype (see below). We concluded from these experiments that point mutation of either Sp/SPLALLA-motif or zinc finger in Elb resulted in dominant-negative function, possibly by sequestering factors present in the DRA (possibly Hth), through the unaltered parts of the over-expressed inactivated protein. The dominant-negative function of over-expressed, point-mutated forms of Elb might therefore result in inactive protein complexes, similar to what has been described for Hth^HM^ [68]. Hence, these results suggested that Sp/SPLALLA domain and zinc finger were crucial for the *in vivo* function of Elb. Interestingly, the identical point-mutations in the Sp/SPLALLA or zinc finger motifs of the Noc protein did not result in a dominant-negative effect on DRA specification (Supplemental Figure S6A–F).

### Mutagenesis of conserved Elb domains interferes with different functions of Orthodenticle (Otd)

Outside the DRA, over-expression of wild type Elb led to the loss of *rh3* and *rh5* expression, while *rh6* was expanded into outer photoreceptors (Supplemental Figure S5A–G). This phenotype, which was observed both in wild type and in *elb,noc* double mutants backgrounds was identical to the loss of Otd function in *otd^UVI^* mutants [49], [50]. Gain-of-function experiments using UAS-*noc* resulted in a similar, but much milder phenotype, with only some outer photoreceptors de-repressing Rh6 (Supplemental Figure S5K,L). Importantly, over-expression of Elb did not repress larval or adult *otd* expression (Supplemental Figure S5I) and expression of *elb*/*noc* was unaltered in *otd^UVI^* mutants (Supplemental Figure S4F,G). Hence, since *elb* ^3.3.1^,*noc^Δ64^* mutant eyes showed no phenotype outside the DRA, we concluded that these gain-of-function phenotypes might be due to an antagonistic genetic interaction between Elb/Noc and Otd, which is expressed in all photoreceptors [69] (although it affects Rhodopsin expression in specific photoreceptor subtypes, see discussion) [49], [50].

Interestingly, the phenotypes observed outside the DRA when over-expressing different point-mutated versions of Elb and Noc separated different aspects of the *otd^UVI^* phenotype:

- Over-expression of *elb*[Gro*] with LGMR-GAL4 resulted in the loss of Rh3 and Rh5 in the entire retina, while expression of Rh4 and Rh6 was normal (Figure 6A–C), leading to ‘empty’ pR7 and pR8 cells. Therefore, while *elb*[Gro*] was still able to antagonize the transcriptional activation of *rh3* and *rh5* by Otd in ‘pale’ ommatidia, Rh6 was not de-repressed in outer photoreceptors. It therefore appears that the putative Groucho-binding motif in Elb is specifically required to genetically antagonize the repressor function of Otd in R1-6, potentially by interfering with its ability to activate expression of the repressor Dve [50].
- Over-expression of *elb*[SPLALLA*] led to a specific loss of Otd's ability to activate both *rh3* and *rh5* expression, but not to repress *rh6* in outer photoreceptors. Therefore, the Sp/SPLALLA motif is specifically required for Elb to antagonize Otd's activator function on *rh3* and *rh5*. Hence, the Gro-binding and Sp/SPLALLA domains therefore specifically affect opposite arms of the interlocked feedforward loops though which Otd and Dve activate or repress *rhodopsin* transcription [50].
- The activator function of Otd could even further be separated though mutation of the Elb zinc finger, since Rh3 expression was normal outside the DRA in LGMR > *elb*[ZnF*] flies, while Rh5 remained lost (See Figure 6D for a table summarizing the effects of wild type, SPLALLA*, Gro*, and ZnF* gain-of-function experiments on DRA specification, as well as the different aspects of *otd* ^UVI^ loss-of-function). Like for the wild type Noc protein, the phenotypes obtained with point-mutated versions of Noc were extremely mild, only affecting the de-repression of Rh6 into outer photoreceptors for *noc*[ZnF*] (Supplemental Figure S6D-F), and *noc*[Gro*] (Supplemental Figure S6G-I). In conclusion, the different aspects of the *otd^UV^* ^I^-like phenotype obtained in an Elb(wt) gain-of-function could be dissociated, by mutating either of the three conserved domains: the Sp/SPLALLA motif, the putative Groucho-binding domain, and the zinc finger.

### VP16 and Engrailed[R] fusions of Noc have a strong effect on R8 Rhodopsin expression

**Figure 7:**
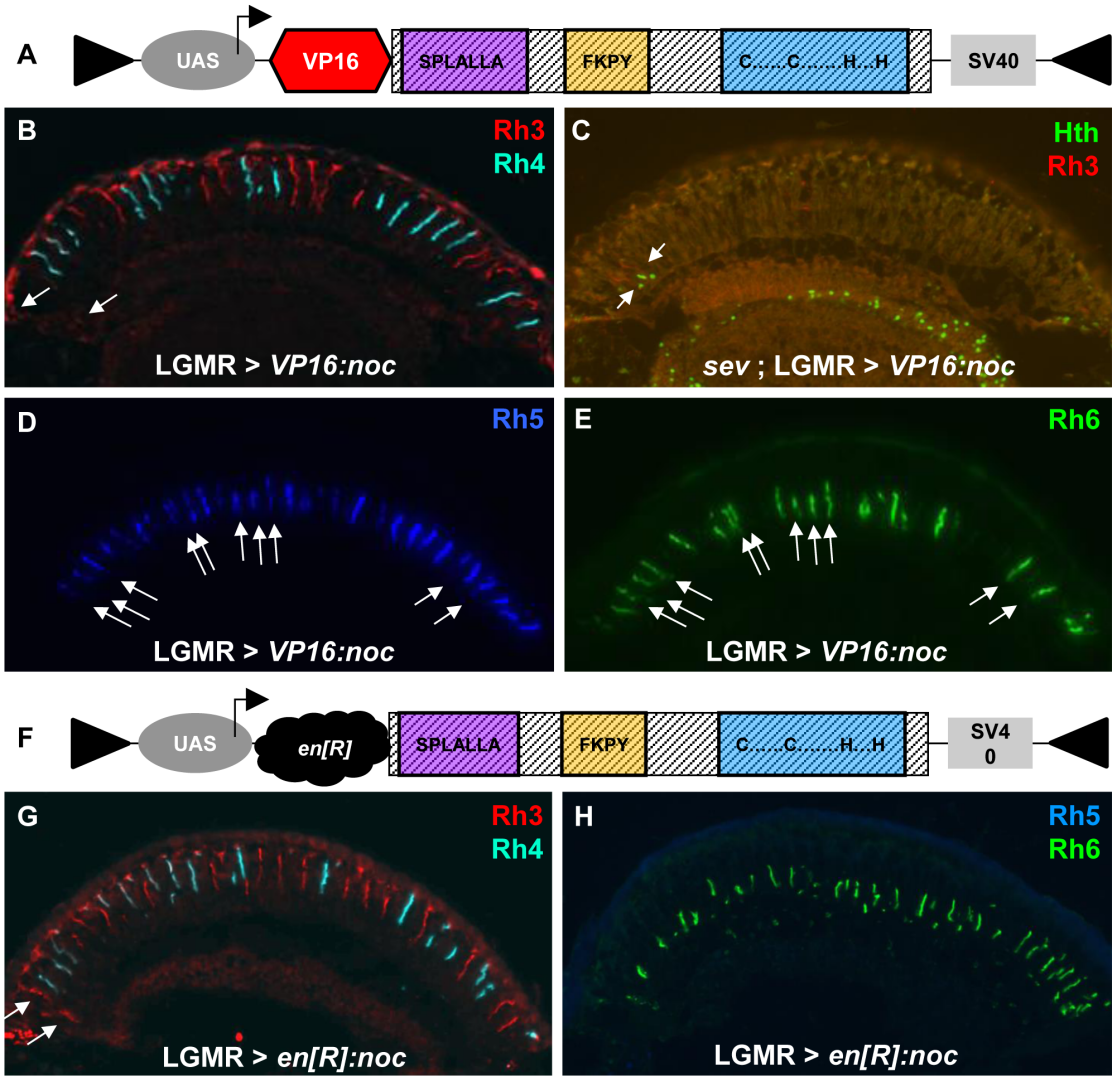
VP16- and *en*[R]-fusions of Noc specifically affect R8 rhodopsin expression. **A.** Schematic of VP16:*noc* fusion cDNA generated in UAS-constructs for over-expression (see Materials and Methods). **B**,**C.** Mild effect of VP16:*noc* over-expression on DRA development: Rh3 expression (red) in the DRA is weak (arrows), yet detectable (B), and Hth expression (green) is normal (C, arrows). **D,E.** VP16:*noc* has a strong activating effect on Rh5 expression (blue), resulting in a high ratio of pR8 cells with Rh5. Since Rh6 expression appears normal (green), many R8 cells now co-express the two R8 rhodopsins (white arrows). **F.** Schematic of Engrailed fusion cDNAs generated for Noc (see Materials and Methods). **G.** Ectopic over-expression of *en[R]:noc* has no effect on R7 rhodopsin expression (Rh3 in red; Rh4 in cyan) and DRA specification (arrows). **H.** Expression of Rh5 (blue) is lost upon over-expression of *en[R]:noc*, and Rh6 (green) is the only R8 rhodopsin remaining.

To further investigate Elb/Noc's role as activators or repressors of transcription, and how they antagonize Otd, we generated fusions with the VP16 transcriptional activation domain [70], as well as the repressor domain of Engrailed [71] (Figure 7). Both kinds of fusion proteins were placed under UAS control for gain-of-function experiments (see Materials and Methods). Over-expression of VP16:*elb* with LGMR-GAL4 led to a severe disruption of retinal morphology (not shown) that was not observed with UAS-VP16:*noc*. We therefore focused our analysis on Noc fusion proteins (Figure 7A–E). Over-expression VP16:*noc* had no effect on R7 Rhodopsins, although Rh3 expression was weak in the DRA (Figure 7B). We confirmed that the DRA markers Hth and Rh3 were correctly expressed in DRA R8 in *sevenless* (*sev*) mutants (Figure 7C), showing that DRA ommatidia were indeed correctly specified. Rhodopsin expression in R8 outside the DRA was severely disrupted: Rh5 was found expanded to most R8 cells (figure 7D), while Rh6 expression appeared normal (figure 7E). The consequence was co-expression of Rh5 and Rh6 in many R8 cells (∼51%). In wild type flies, such co-expression is prevented by mutually exclusive genes in p and y R8 [72], [73]. We therefore concluded that VP16:*noc* had a direct activating effect on *rh5*, without affecting any other rhodopsin.

Over-expression of the repressor fusion *en[R]:noc* (Figure 7F) also had no effect on DRA specification (Figure 7G). However, expression of Rh5 was lost, while Rh3, Rh4, and Rh6 appeared normal in the rest of the retina (Figure 7H). This R8 phenotype observed with *en[R]:noc* was therefore the opposite of that observed with the activator fusion VP16:*noc*, with both Noc fusion proteins specifically affecting *rh5* expression in opposite manner. Since Otd directly activates *rh3 and rh5* transcription [49], [50], Noc might function as a direct antagonist of Otd's function in R8 cells, but not in R7. Interestingly, we found strong Elb/Noc expression outside the DRA only in R8 cells (Figure 1E–I; Supplemental Figure S1D–G).

Mutations in the human Elb/Noc homolog ZNF703 promote metastasis in luminal breast cancer [22], [23], [74]. To investigate if NET family protein functions are evolutionarily conserved, we generated UAS-constructs for over-expression of both human NET family proteins in *Drosophila* (UAS-ZNF503, UAS-ZNF703; see Materials & Methods). Gain-of-function phenotypes in the retina were very weak, but Rh5 was expanded in R8 cells, leading, in both cases, to co-expression with Rh6 (Supplemental Figure S7A–E). The *C. elegans* homolog TLP-1 was not active in this assay (Supplemental Figure S7F-H). This co-expression phenotype therefore resembles most closely what we had observed for the over-expression of a VP16:*noc*, suggesting that genetic interaction of NET family proteins with Otd/Otx proteins is a conserved feature of these factors.

## Discussion

**Figure 8:**
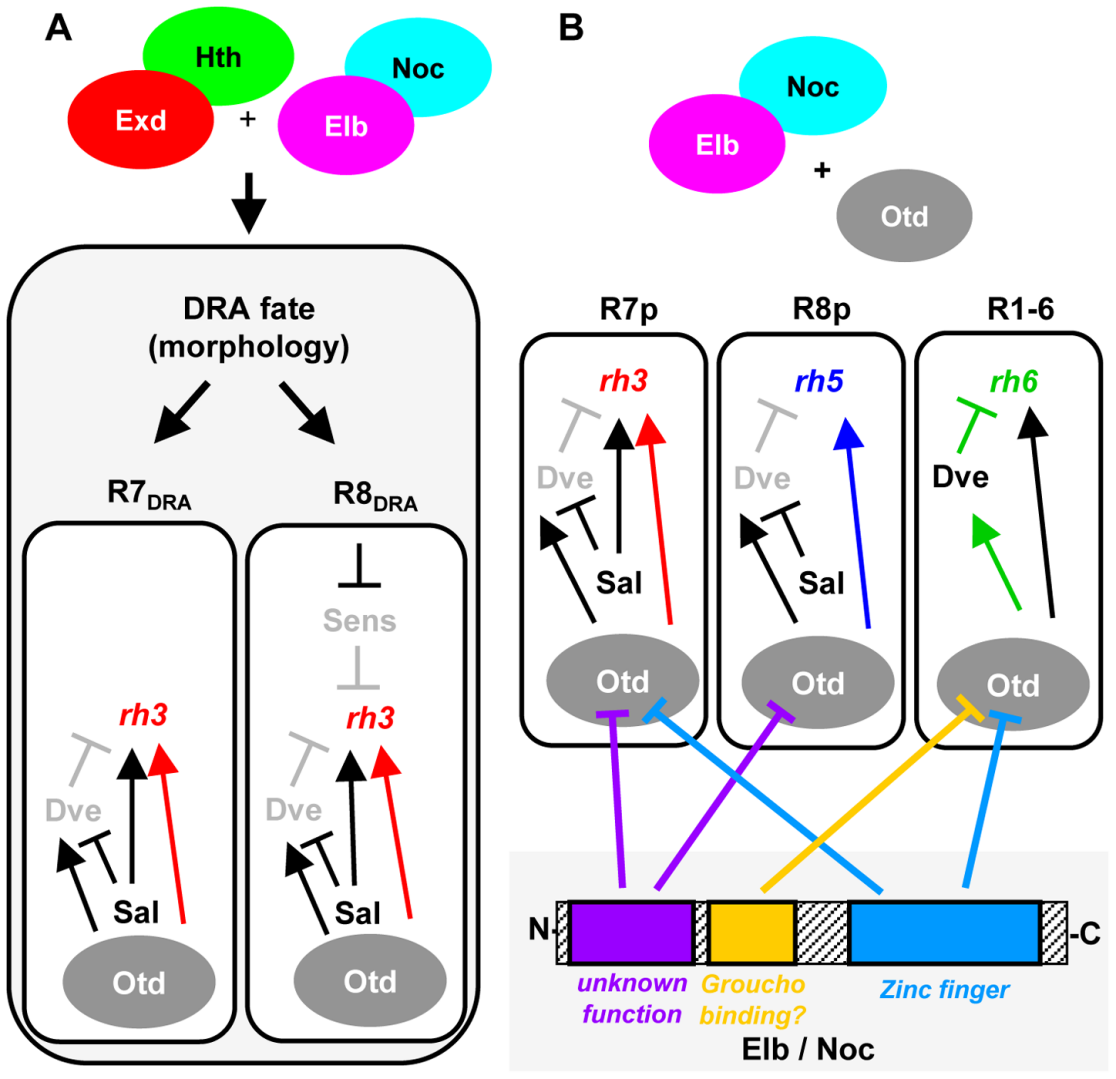
Model summarizing the roles of Elb/Noc action in photoreceptor cell fate specification. Model summarizing the role of Elb and Noc proteins in terminal specification of photoreceptor cell fates. **A.** Role of Elb and Noc in DRA ommatidia. Loss of function data suggests Elb & Noc function with Hth in specifying the DRA cell fates, both morphologically (rhabdomere diameter) and molecularly: repression of Sens in R8_DRA_ by Hth/Exd and Elb/Noc is crucial for these cells to express the DRA rhodopsin marker Rh3. **B.** Dissection of Otd functions through antagonism with Elb/Noc protein domains. The similarity of *elb*/*noc* gain-of-function phenotypes to the previously published *ocelliless* (otd) loss-of-function suggests Otd/Elb/Noc proteins interact genetically. The different transcriptional effects of Otd on *rh3* and *rh5* expression (activation in ‘pale’ ommatidia), as well as *rh6* (repression via Dve in outer photoreceptors) can be neutralized independently, using different mutated forms of Elb/Noc. The specific role of three conserved Elb/Noc domains (Sp/SPLALA, Gro-binding, Zn finger) in genetically antagonizing Otd function are indicated by inhibition arrows.

The transcription factors Homothorax (Hth) and Extradenticle (Exd) have been well characterized as co-factors for Hox genes [48]. Hth/Exd can also act as co-factors for non-Hox transcription factors, like for Engrailed [75], [76]. Here we showed that loss of both Elb and Noc phenocopies the loss of Hth at the dorsal rim of the retina. All markers of DRA ommatidia are lost in *elb,noc* double mutants: Rh3 expression and Sens repression in DRA R8, as well as the DRA-specific inner photoreceptor rhabdomere morphology in DRA R7 and DRA R8. Our data shows that Elb/noc act downstream of Hth in the specification of DRA cell fates. Elb and Noc are expressed strongly in DRA R7 and R8. This expression is expanded to all R7 and R8 by ectopic Hth (but never into outer photoreceptors R1-6), while Hth expression is not affected in *elb,noc* double mutants. One possibility is that Elb/Noc serve as cofactors for Hth/Exd (Figure 8A), since Hth loses its potential to induce the DRA fate in a double mutant retina. The vertebrate homologs of Elb and Noc function as repressors of transcription [13]. Therefore, aspects of the Hth/Exd and Elb/Noc loss-of-function phenotypes could be due to a direct failure of their complex to repress common target genes. For instance, the de-repression of the R8 marker Sens by dominant-negative hth^HM^, as well as in *elb,noc* double mutants could be explained by loss of a repressor complex containing all four proteins. Interestingly, functional antagonism between the Hox/Hth/Exd complex and Sens have been described in the *Drosophila* embryo [77]. However, in this case the factors were shown to compete for overlapping binding sites in the promoter of the common target gene *rhomboid*. Gene expression profiling data revealed that the Hox gene Abd-B also directly represses Sens in the embryo using Hth/Exd as cofactors [78]. Elb and Noc might therefore provide a missing link for transcriptional repression of Sens by Hth/Exd.

Much work on NET family proteins has focused on functional characterization of their evolutionarily conserved domains. The C-terminus of NET proteins is required for nuclear localization [14], [79], as well as for self-association of the zebrafish ortholog Nlz1, although neither self-association nor heterodimerization with Nlz2 was found to be necessary for wild type function [79]. The ‘buttonhead box’ [80], a conserved 7–10 amino acid motif which we have not investigated in this study, may be required for transcriptional activation [81]. Deletion of the ‘buttonhead box’ in zebrafish Nlz proteins transformed them into dominant-negatives, an effect that was proposed to be due to reduced affinity to co-repressor Groucho and histone de-acetylases [12], [79]. Interestingly, deletion of N-terminal sequences, including the Sp/SPLALLA motif also leads to dominant negative proteins [79]. These data are consistent with our findings that a protein with a mutated Sp/SPLALLA motif has a dominant-negative effect on DRA specification. The Sp motif was proposed to mediate transcriptional repression by directly binding to cofactors [15]. It should be noted that both N-terminal Sp/SPLALLA deletion and the VP16 fusions have the same dominant-negative effect for zebrafish Nlz1 [12]. While this is consistent with a pure repressor function of the zebrafish protein, the differences between Sp/SPLALLA mutation and VP16-fusion (as well as the observation of a phenotype for the Engrailed fusion) reported in this study hint towards a more complex role of Elb and Noc in transcriptional regulation.

We showed that mutation of the conserved zinc finger of Elbow also transforms this protein into a dominant-negative. Usually, multiple zinc fingers are required for DNA binding, suggesting that the NET family zinc finger is a protein-protein interaction domain [11], [67]. Deletion of the zinc finger from zebrafish Nlz proteins leads to a loss of nuclear localization [79], and the Nlz1 zinc finger is necessary for transcriptional repression [13]. Although we cannot exclude the possibility that Elb and Noc bind DNA through their zinc finger, it is likely that mutation of the zinc finger either leads to an inactive complex by sequestration of another co-repressor, or that such complex could be trapped in the cytoplasm. Given that mutation of either Sp/SPLALLA motif or zinc finger both lead to a dominant-negative effect raises the possibility that protein binding to both motifs could be necessary for *in vivo* function, possibly through the formation of higher order transcriptional complexes.

Loss of both *elb* and *noc* does not result in Rhodopsin phenotypes outside the DRA. However, over-expression of different forms of Elb or Noc recapitulates all Rhodopsin phenotypes observed in *otd^UVI^* mutants [49], [50]. This phenotype might therefore arise from forcing a direct interaction between over-expressed Elb protein and Otd. Little is known about the regulatory relationship between Elb/Noc and Otd. However, the overlapping expression patterns and similar phenotypes for certain alleles of *otd* named *ocelliless*, and for *no ocelli* (*noc*) at the anterior pole of the fly embryo, as well as their common requirement in the morphogenesis of ocelli suggests that these proteins also interact positively outside of the retina. The antagonism we observed might therefore be a dominant-negative effect resulting from sequestration of the Otd protein by over-expressed Elb. Alternatively, different combinations of transcriptional cofactors present between tissues (for instance DRA versus non-DRA R8 cells) might decide whether Elb and Noc act in concert with Otd, or as antagonists.

In the retina, Otd acts in a ‘coherent feedforward loop’ with Spalt to directly activate transcription of *rh3* and *rh5* [50]. As a consequence, Rh3 and Rh5 are lost in *otd* mutants. Furthermore, Otd activates transcription of the repressor Dve, forming an ‘incoherent feedforward loop’, resulting in repression of *rh3* and *rh5* in outer photoreceptors. Since *rh6* is activated by a distinct factor, Pph13 [51], loss of Otd leads to a specific de-repression of *rh6* into outer photoreceptors [50]. We show that different domains of Elb specifically interfere with different aspects of Otd function in these feedforward loops (Figure 8B). Mutation of the Groucho-binding motif FKPY only abolishes the ability of over-expressed Elbow protein to antagonize Otd function in repressing *rh6* in outer photoreceptors, while mutation of the Sp/SPLALLA motif specifically antagonizes Otd function in activating both *rh3* and *rh5*, without affecting repression of *rh6* in outer photoreceptors (mediated by induction of Dve). Furthermore, while the Elb zinc finger is also required for antagonizing the function of Otd in outer photoreceptors, it is also necessary for antagonizing activation of *rh3* by Otd, but not *rh5*. Hence, these two activator functions of Otd could be separated by mutating the zinc finger.

The different Rhodopsin phenotypes caused by loss of Otd can be mapped to different protein domains [51]. Our data therefore reveal specific genetic interactions between the protein domains of Elb/Noc and Otd. Such interactions could be direct or be mediated through additional proteins. For instance, the Otd C-terminus mediates the repression of *rh6* in outer photoreceptors [82], making it a possible interaction domain for Groucho binding to the Elb/Noc FKPY motif. The N-terminus of Otd is necessary for most activation potential on *rh3*, while activation of *rh5* predominantly maps to the C-terminus [82]. This correlates well with the Rhodopsin-specific phenotypes we see after mutation of Sp/SPLALLA (affecting *rh3* and *rh5*), or the zinc finger (affecting *rh3 and rh6*) motifs. Finally, our results using VP16- and en[R]-fusions of Noc show that potentially direct transcriptional effects on rhodopsin genes can only be induced in R8 cells. Both fusion proteins specifically regulate expression of *rh5*, while all other rhodopsins remain unaffected. Elb and Noc are both expressed strongly in R8 cells outside of the DRA where they may contribute the repression of Rh5. The absence of a non-DRA R8 rhodopsin phenotype in *elb,noc* double mutants, as well as the R8-specific action of VP16:*noc* could therefore be due to the existence of redundant, R8-specific factors required for Elb/Noc function there, but not for DRA specification. These factors remain unknown, since we found that expression of *elb* and *noc* is not altered in homozygous mutants affecting p/y cell fate decisions in R8 cells (*melt* and *wts*, [72]; Supplemental Figure S8, A–D), like in R7 cells (Supplemental Figure S8E,F).

Mutations in the human Elb/Noc homolog ZNF703 promote metastasis [6]. We have shown that over-expression of both human NET family proteins UAS-ZNF503 and UAS-ZNF703 in the *Drosophila* retina result in weak co-expression of Rh5 and Rh6, resembling over-expression of a VP16:*noc* protein. It is therefore possible that the genetic interaction of NET family proteins with Otd/Otx proteins is evolutionarily conserved, especially since a central domain of Otd was previously shown to mediate mutual exclusion of Rh5 and Rh6 [82]. Here we present a new role for *Drosophila* NET proteins in retinal patterning. Both zebrafish homologs of Elb/Noc, Nlz1 and Nlz2 are also required for optic fissure closure during eye development [83]. Furthermore, expression of the Elb/Noc mouse homologue znf503 suggests that NET family genes are involved in the development of mammalian limbs [84]. Given previous reports from *Drosophila* on the proximo-distal specification of leg segments [24], it appears that NET family members act in similar processes across species. This raises the possibility that NET proteins serve as evolutionarily conserved modules that have been re-utilized for analogous processes during evolution. Based on our data, their conserved domain structure might be crucial for interacting with transcription factor networks involving conserved families of factors like Otx or Meis. Given their medical relevance in breast cancer, a better understanding of the role NET proteins play in the transcriptional control of tissue patterning will be of great importance.

## Materials and Methods

### Fly stocks

- GAL4 drivers: *elb*-GAL4 and *noc*-GAL4 [24], LGMR-GAL4 [2], [85].
- UAS-constructs: UAS-*elb* (U. Weihe & S.M. Cohen), UAS-*noc* (U. Weihe & S.M. Cohen), UAS-VP16:*elb* (this study), UAS-VP16:*noc* (this study), UAS-*en[R]:elb* (this study), UAS-*en[R]:noc* (this study), UAS-*elb*[SPLALLA*] (this study), UAS-*noc*[SPLALLA*] (this study), UAS-*elb*[Gro*] (this study), UAS-*noc*[Gro*] (this study), UAS-*elb*[ZnF*] (this study), UAS-*noc*[ZnF*] (this study), UAS-TLP-1 (this study), UAS-ZNF703 (this study), UAS-ZNF503 (this study), UAS-GFP:*hth* (R. Mann), UAS-*myc:hth* (R. Mann), UAS-GFP:*hth^HM^* (R. Mann), UAS-*arm^S10^* [64], UAS-arm*^Δ^* ^N^ (F. Pichaud), UAS-*lacZ* (J. Treisman), UAS-lacZ:NLS (Bloomington Stock Center), UAS-eGFP (M. Wernet), UAS-GFP:NLS (Bloomington Stock Center).
- p{PZ} enhancer traps: *hth^l^* ^(3)06762^-PZ/TM3 (Bloomington stock center), *svp*-PZ/TM3 (U. Gaul).
- clonal analysis: *ey*-Flip (B. Dickson), FRT40-*elb,noc*[64-1-4][24], FRT40- noc*^Δ^*64 [10], FRT40-*arm*-lacZ (J. Treisman), FRT40-p[w+] (J. Treisman), FRT82B-*arm*-lacZ (F. Pichaud), FRT82B-*ss^D115.7^* (I. Duncan), FRT82B-*wts^P1^*/TM2 (T. Xu), *melt* ^32.1a^ (S. Cohen).
- Viable mutants: *elb^3^* ^.3.1^ (U. Weihe), *elb^3^* ^.3.4^ (U. Weihe), *otd^UVI^* (R. Reinke).
- other: LGMR-GFP:*hth* (this study), *rh1*-lacZ, *rh3*-lacZ, *rh4*-lacZ, *rh5*-lacZ, *rh6*-lacZ (Bloomington stock center), GMR-arm* [65]

### Molecular biology

#### Generation of LongGMR-GFP:*hth* transgenes

The ORF of the GFP:*hth* fusion protein was excised from pUAST-GFP:*hth* (gift from R. Mann, Columbia University) using Xba1, and ligated into a pre-existing pCasper4-LongGMR-mcs-SV40 vector (B. Mollereau, M. Wernet, and C. Desplan, unpublished). Sequence available upon request.

#### Generation of VP16 and En[R] fusions

A ∼500 bp 5′ fragment of *elb* was amplified from the full-length cDNA [10] replacing the start ATG with an EcoR1 site (altering the amino acid sequence from MLQ to EFLG). This fragment was digested EcoR1/Xho1(489) and triple ligated into pVP16 (Clontech Laboratories Inc.) digested (EcoR1/Hind3), using an Xho1/Hind3 fragment providing the rest of the *elb* cDNA. The same strategy was used for VP16:*noc*: the start ATG of *noc* was replaced with an EcoR1 site (MVV → EFVV). The resulting ∼650 bp fragment was digested (EcoR1/Bgl2) and triple-ligated into pVP16 (EcoR1/Xho1), using a (Bgl2/Xho1) fragment from the full-length noc cDNA [10]. The VP16 fusions were then removed from pBSK (Bgl2/Xba1) and ligated into pUAST [86].

A 892 bp 5′ fragment from *engrailed* (*en*) containing its repressor domain as PCR-amplified from a full length clone (gift from T. Cook), introducing an EcoR1 site 5′ of the ATG. In parallel, the ATG's of both *elb* and *noc* [10] were then replaced with BamH1 sites (GGATCC). The En repressor domain was then digested EcoR1/BamH1 and ligated into pBSK (EcoR1/Xho1), together with a (BamH1/Xho1) fragment providing the rest of the *elb* or *noc* coding sequence, respectively. Fusion cDNAs were sequenced and subcloned from pSK into pUAST (EcoR1/Xho1).

#### Site–directed mutagenesis of SPLALLA, Gro, and ZnF motifs in *elb* and *noc*

a) Site-directed mutagenesis of conserved SPLALLA motifs in both Elb and Noc: The amino acid sequence of this motif was altered to TGIVIIV in both proteins, using overlapping PCR primers with an altered nucleotide sequence (AGT CCG TTG GCG CTA TTG GCC → ACT GGG ATT GTG ATA ATC GTC for *elb*, and AGT CCC TTG GCT CTG CTC CTA → ACT GGC ATT GTT ATT ATC GTA for *noc*). The resulting ∼500 bp mutant fragment was digested Spe1/Xho1 (*elb*), or Hind3/Bgl2 (*noc*), and ligated into pBSK together with a second fragment providing the rest of the respective cDNA (Xho1/Hind3 for *elb*, and Bgl2/Xho1 for *noc*).

b) Site-directed mutagenesis of conserved Groucho-binding motifs in both Elb and Noc: The amino acid sequence was altered to IEGS in both proteins, using overlapping PCR primers (TTT AAG CCC TAC → ATT GAG GGC TCC for *elb*, and TTC AAG CCC TAC → ATC GAG GGC TCC for *noc*). The resulting mutant fragment was digested Nco1 (*elb*, ∼600 bp), or Bgl2/Mlu1 (*noc*, ∼210 bp), and ligated into pBSK-*elb* (digested Nco1), or pBSK-*noc* (digested Bgl2/Mlu1), respectively.

c) Site-directed mutagenesis of conserved zinc fingers in both Elb and Noc: The two Histidines in both proteins were altered to Alanines, using overlapping PCR primers (CAT CTG CGC ACC CAT → GCT CTG CGC ACC GCT for *elb*, and CAT CTG CGC ACC CAT → GCT CTG CGC ACC GCT for *noc*). The resulting mutant fragment was digested BsiW1 (*elb*, resulting fragment size: ∼220 bp), or Nco1/Sac2 (*noc*, ∼420 bp), and ligated into pBSK-*elb* (digested BsiW1), or pBSK-*noc* (digested Nco1/Sac2), respectively.

Finally, all mutated cDNAs were sequenced and then subcloned (EcoR1/Xho1) from pBSK into pUAST [86].

#### Generation of UAS-TLP-1, UAS-ZNF503, and UAS-ZNF703

The entire ORF of *C. elegans* TLP-1 was PCR amplified from a full-length cDNA clone (gift from D. Fitch, NYU), using restriction sites attached to the primers: EcoR1 (5′), and Xho1 (3′). The product was digested EcoR1/Xho1 and ligated into pUAST [86]. The ORF's of human homologues ZNF503 and ZNF703 were excised EcoR1/Xho1 from full-length clones MGC2555 (image:3604473) and FLJ14299 (image:5527569), respectively (from ATCC Inc.) and ligated into pUAST (digested EcoR1/Xho1).

### Immunohistochemistry

Primary antibodies used were anti-βGal rabbit polyclonal 1/5000 (Cappel), anti-βGal mouse monoclonal 1/500 (Promega), anti-Homothorax guinea pig polyclonal 1/500 (R. Mann, Columbia University), anti-ElaV mouse or rat monoclonals 1/10 (Iowa University Hybridoma bank), anti-24B10 mouse monoclonal 1/10 (Iowa University Hybridoma bank), anti-Prospero mouse monoclonal 1/4 (Iowa University Hybridoma bank), anti-Senseless guinea pig polyclonal 1/10 (H. Bellen, Baylor College), anti-Rh3 mouse monoclonal 1/100 (S. Britt, University of Colorado), anti-Rh3 chicken polyclonal 1/20 (T. Cook, University of Cincinnati), anti-Rh4 mouse monoclonal 1/100 S. Britt), anti-Rh5 mouse 1/100 (S. Britt, anti-Rh6 rabbit polyclonal 1/1000 [49].

Secondary antibodies were a) AlexaFluor488 coupled made in goat or donkey, anti-rabbit, mouse, rat or guinea pig (Molecular Probes), b) Cy3 or TxRed-coupled made in goat or donkey, anti-rabbit, mouse, rat, guinea pig or chicken (Jackson Immunochemicals) and c) Cy5 coupled made in goat or donkey, anti-mouse or rat (Jackson Immunochemicals).

#### Pupal dissections

Pupal retinas were staged and dissected essentially as previously described [2], were fixed with 4% formaldehyde for 25 min and washed with PBS+0.3% Triton X-100. Incubation with primary antibodies was performed at 4°C overnight in BNT [PBS(1x), 250 mM NaCl, 1% BSA, 1% Tween 20], and secondary antibodies (1/200 in BNT) were applied for at least 2 hours at RT.

#### Adult cryostat sections

All used transgenic constructs were crossed into a *cn bw* background [38] to eliminate eye pigmentation. Frozen sections of adult heads were performed using a cryostat microtome (Zeiss) and deposited on superfrost Plus slides (Fisher), as previously described [43]. For immuno Histochemistry conditions were the same as above. For X-Gal reactions, Horizontal eye sections were fixed 5±10 min in PBS 0.25% gluteraldehyde. They were stained in a solution of 7.2 mM Na2HPO4, 2.8 mM NaH2PO4, 150 mM NaCl, 1 mM MgCl2, 3 mM K3[Fe(CN)6], 3 mM K4[Fe(CN)6], containing a 1/30 dilution of X-Gal (30 mg/ml in dimethyl formamide). After washing in PBS, slides were mounted in aquamount (Lerner Laboratories, Fisher).

#### Adult plastic sections

For the morphological examinations with transmission light microscopy, the eyes were fixed with 2% glutaraldehyde (sometimes plus 1% OsO_4_) in 0.05 M Na-cacodylate buffer (pH 7.2) for 2 h at 4°C. Following post-fixation with 2% OsO_4_ in 0.05 M Na-cacodylate buffer (pH 7.2) for 2 h at 4°C, the tissue was dehydrated with 2,2-dimethoxypropane and embedded in Epon 812. 1 µm sections for light microscopy were stained with methylene blue.

#### Imaging software

All fluorescent microscopy was performed using a Nikon Microphot-SA and super high pressure mercury lamps (Hg 100 watts, Ushio Electric). Confocal microscopy was performed using a Leica TCS S2 system. Digital images were produced using SPOT software.

## Supporting Information

Figure S1 Additional expression data for *elb* and *noc.* **A**. Larval expression of *elb*-GAL4 in the 3^rd^ instar eye-antennal disc: strong expression can be seen in the antennal disk, as well as anterior to the morphogenetic furrow (MF; white arrow head). Posterior to the MF, strong expression becomes visible in two photoreceptors per ommatidium, identified as R3 and R4, by co-staining with Spalt (red; in the first few ommatidial rows expressed in R3&R4, then later in R7&R8). See A′ for magnification. **B**. Expression of *elb*-GAL4 in 3^rd^ instar eye discs, labeled with UAS-GFP:NLS. Double labeling with *svp*-lacZ:NLS identifies labeled cells as R3 an R4 (see magnification; inset). **C.** During pupal development, *elb* becomes expressed in R7 and R8 (labeled with Spalt, red). **D**,**E.** Expression levels of *elb*-GAL4 vary strongly between cells. Some R7 cells express *elb* very strongly, while others appear almost void of staining (D; co-labeled with Pros, green). The same is true for R8 cell expression, co-labeled with Sens (green) (E). **F**,**G.** Different expression levels in R8 cells do not correlate with opsin subtypes. Double labeling of βGal and Rh5 (E), or Rh6 (F) do not reveal any systematic correlation.
(TIF)

Figure S2 *elb*, *noc* single, or double mutants have no phenotype outside the DRA. **A**,**B**. Central photoreceptor opsin expression in the wild type retina: R7 opsins Rh3 (red) and Rh4 (cyan) (A), and R8 opsins Rh5 (blue) and Rh6 (green) labeled on Cryostat cross section (B). **C**,**D.** Opsin expression in R7 and R8 cells is normal in *elb^3.3.1^* (-/-) single mutants: R7 and R8 in the DRA (dashed white box) are labeled with Rh3 (red), while Rh4 (cyan) in yR7 cells outside the DRA is normal (C). p/y opsin ratios appear normal in both R7 and R8 (D). **E**,**F.** No change in opsin expression is visible in homozygous noc^Δ64^ (-/-) mutant flies. **G**,**H.** Coupling of central photoreceptor opsins is normal in *elb^3.3.1^,noc ^Δ64^* (-/-) double mutants: coupled expression of Rh3 (red) and Rh6 (green) within the same ommatidium is not observed. The same is true for coupling of Rh4 (yellow) and Rh5 (blue). Positively labeled R7 cells are always coupled with gaps in the R8 opsin pattern, as reported for wild type flies.
(TIF)

Figure S3 Photoreceptor specification is normal in *elb*,*noc* double mutants. **A.** During pupation, DRA ommatidia labeled with Exd (red) are specified normally in *elb^3.3.1^,noc ^Δ64^* (-/-) double mutant clones (marked by the absence of *arm*-*lacZ*, green). **B.** Similarly, R8 cells are specified correctly, as seen with Sens (green) being unaltered in double mutant clones (marked by the absence of *arm*-*lacZ*, red). **C**,**D.** Both situations remain indistinguishable from wild type flies throughout adulthood. **E.** Specification of inner photoreceptors R7 is normal in *elb^3.3.1^,noc ^Δ64^* double mutant clones; labeled: Pros (green) and *arm*-*lacZ* (red). **F.** Expression of inner photoreceptor marker Spalt (red) is unaltered inside homozygous *elb.noc* clones.
(TIF)

Figure S4 *elb, noc, and hth* expression in different genetic backgrounds. **A.** Schematic of LGMR-GFP:Hth transgenes generated for this study. **B.** In the adult eye, Hth (green) is over-expressed in all photoreceptors, by the direct fusion transgene LGMR:GFP:Hth, and expression of *elb*-GAL4 is not expanded (visualized using UAS-lacZ:NLS, red). **C.** The entire eye is transformed into DRA ommatidia, as previously shown using the GAL4/UAS technique: Rh3 (red) is expanded throughout the retina. Rh4, Rh5, and Rh6 are lost (not shown). **D**,**E.** Ectopic activation of the wingless pathway (LGMR-arm*) leads to an expansion of Rh3/Rh3 coupled DRA ommatidia (schematic, left) across the dorsal half of the retina (dashed white box), with Rh6 and Hth expression excluding each other. **E.** Co-expression of Hth and Rh6 in the dorsal eye in *elb,noc* double mutants, when the Wingless pathway is ectopically activated using LGMR-arm*, resulting in ‘odd-coupled’ Rh3/Rh6 ommatidia (schematic, left). **F**,**G.** Expression of *elb*-GAL4 (red) is unchanged in eye-specific mutants loss-of-function mutants of *otd* (*ocelliless, oc*), called *otd^UVI^*. Note expansion of Rh6 expression (green) into outer photoreceptors, in these mutants (G), with Rh6-positive rhabdomeres spanning the entire thickness of the retina.
(TIF)

Figure S5 Gain-of-function phenotypes of *elbow* and *no ocelli.* **A.** Schematic showing structure of UAS-*elb* transgenes used for mis-expression of wild type Elbow protein. **B–G.** The gain-of-function opsin phenotype obtained with UAS-*elb* phenocopies *otd^UVI^* mutants [49]. Wild type expression (X-Gal staining on Cryostat cross-sections) of *rh1*-lacZ (B), *rh3*-lacZ (C), and *rh6*-lacZ (D). Expression of *rh3*- and *rh5*-lacZ was completely lost in LGMR > *elb* flies (E, F). Expression of *rh6*-lacZ was expanded into outer photoreceptors (G), as seen by labeled projections into the lamina (black arrow). Expression of *rh1*- and *rh4*-lacZ was normal (not shown). **H.** Eye phenotype obtained when over-expressing elbow in all photoreceptors, using LGMR-GAL4: the compound eye gets shiny and slightly rough. **I.** Over-expression of wild type Elb protein does not repress transcription of *oceliless* (Otd). Expression of *oc*-lacZ (green) is not affected in eye imaginal discs of LGMR > *elb* flies, double-labeled with Anti-ElaV (red, I′). **J.** Schematic of UAS-*noc* transgenes used. **K**,**L.** The *no ocelli* gain-of-function opsin phenotype is weak: While all other opsins are expressed normally, only *rh6* shows a weak expansion into outer photoreceptors, as seen with *rh6*-lacZ (K; black arrow) and Anti-Rh6 signals (green) (L, white arrows) spanning the entire thickness of the adult retina.
(TIF)

Figure S6 Over-expression of mutated forms of No ocelli. **A.** Schematic of UAS-transgene generated for mis-expression of Noc protein with a mutated Sp/SPLALLA motif (see Materials and Methods). **B.** DRA specification and R7 opsin expression is not affected by ectopic Noc[SPLALLA*]. **C.** R8 opsin expression is mildly affected: Rh6 expression (green) is expanded into some outer photoreceptors (white arrow), while Rh5 expression (blue) is normal. **D.** Schematic of UAS-transgene generated for mis-expression of No ocelli protein with a mutated Groucho-binding motif (FKPY → IEGS; see Materials and Methods). **E+F.** DRA specification and inner photoreceptor expression are not affected by ectopic over-expression of Noc[Gro*]. **G.** Schematic of UAS-transgene generated for mis-expression of Noc protein with a mutated zinc finger, where both zinc finger Histamines have been mutated to Alanines (see Materials and Methods). **H**, **I.** DRA specification and inner photoreceptor expression are not affected by ectopic over-expression of Noc[ZnF*].
(TIF)

Figure S7 Over-expression of worm and human homologues of Elb/Noc. **A.** Schematic of UAS-transgene generated for mis-expression of the human homologues of Elb/Noc: ZNF703 and ZNF503. **B**,**D.** DRA specification and R7 opsin expression are not affected by ectopic over-expression of ZNF703 (B), or ZNF503 (D). **C**,**E.** R8 opsin expression is mildly affected by ectopic over-expression of ZNF703 (C), or ZNF503 (E): occasional co-expression of Rh5 (blue) and Rh6 (green) is observed (white arrows), for both homologues. **F.** Schematic of UAS-transgene generated for mis-expression of the *C. elegans* homologue TLP1. **G**,**H.** DRA specification and inner photoreceptor expression is not affected by ectopic over-expression of UAS-TLP1.
(TIF)

Figure S8 *elb, noc* expression is normal in *warts*, *melted*, and *spineless* mutants. **A**,**B.** Expression of *elb*-GAL4 (red) is not affected in homozygous mutants for *warts (Dlats, wts)*. Note how expression of pR8 opsin Rh5 (blue) is expanded in these mutants. **C**,**D.** Expression of *elb*-GAL4 (red) is not altered in homozygous mutants for *melted (melt)*. Note how expression of yR8 opsin Rh6 (green) is expanded in these mutants. **E**,**F.** Expression of *elb*-GAL4 (green) is not altered in homozygous *spineless* (*ss*) mutants. Note how expression of pR7 opsin Rh3 (red) is expanded in these mutants, while yR7 opsin Rh4 is lost.
(TIF)
