# Supplementary figures and images for: Genetic Dissection of Photoreceptor Subtype Specification by the Drosophila melanogaster Zinc Finger Proteins Elbow and No ocelli
Source: PLoS Genet. 2014 Mar 13;10(3):e1004210. doi: 10.1371/journal.pgen.1004210 (PMC3953069; doi:10.1371/journal.pgen.1004210)

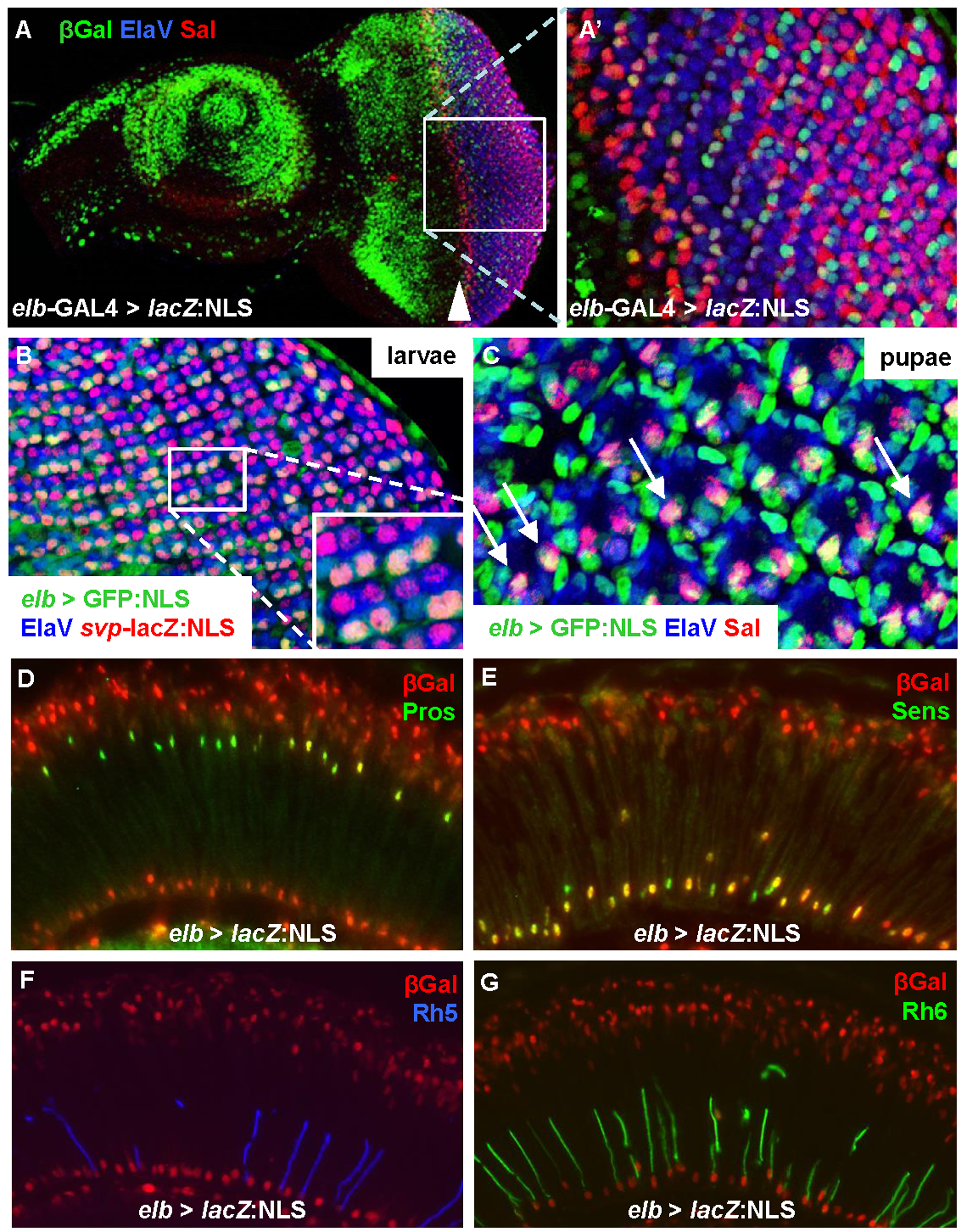

Supplement: Figure S1 — Additional expression data for elb and noc. A. Larval expression of elb-GAL4 in the 3rd instar eye-antennal disc: strong expression can be seen in the antennal disk, as well as anterior to the morphogenetic furrow (MF; white arrow head). Posterior to the MF, strong expression becomes visible in two photoreceptors per ommatidium, identified as R3 and R4, by co-staining with Spalt (red; in the first few ommatidial rows expressed in R3&R4, then later in R7&R8). See A′ for magnification. B. Expression of elb-GAL4 in 3rd instar eye discs, labeled with UAS-GFP:NLS. Double labeling with svp-lacZ:NLS identifies labeled cells as R3 an R4 (see magnification; inset). C. During pupal development, elb becomes expressed in R7 and R8 (labeled with Spalt, red). D,E. Expression levels of elb-GAL4 vary strongly between cells. Some R7 cells express elb very strongly, while others appear almost void of staining (D; co-labeled with Pros, green). The same is true for R8 cell expression, co-labeled with Sens (green) (E). F,G. Different expression levels in R8 cells do not correlate with opsin subtypes. Double labeling of βGal and Rh5 (E), or Rh6 (F) do not reveal any systematic correlation. (TIF) [file pgen.1004210.s001.tif]

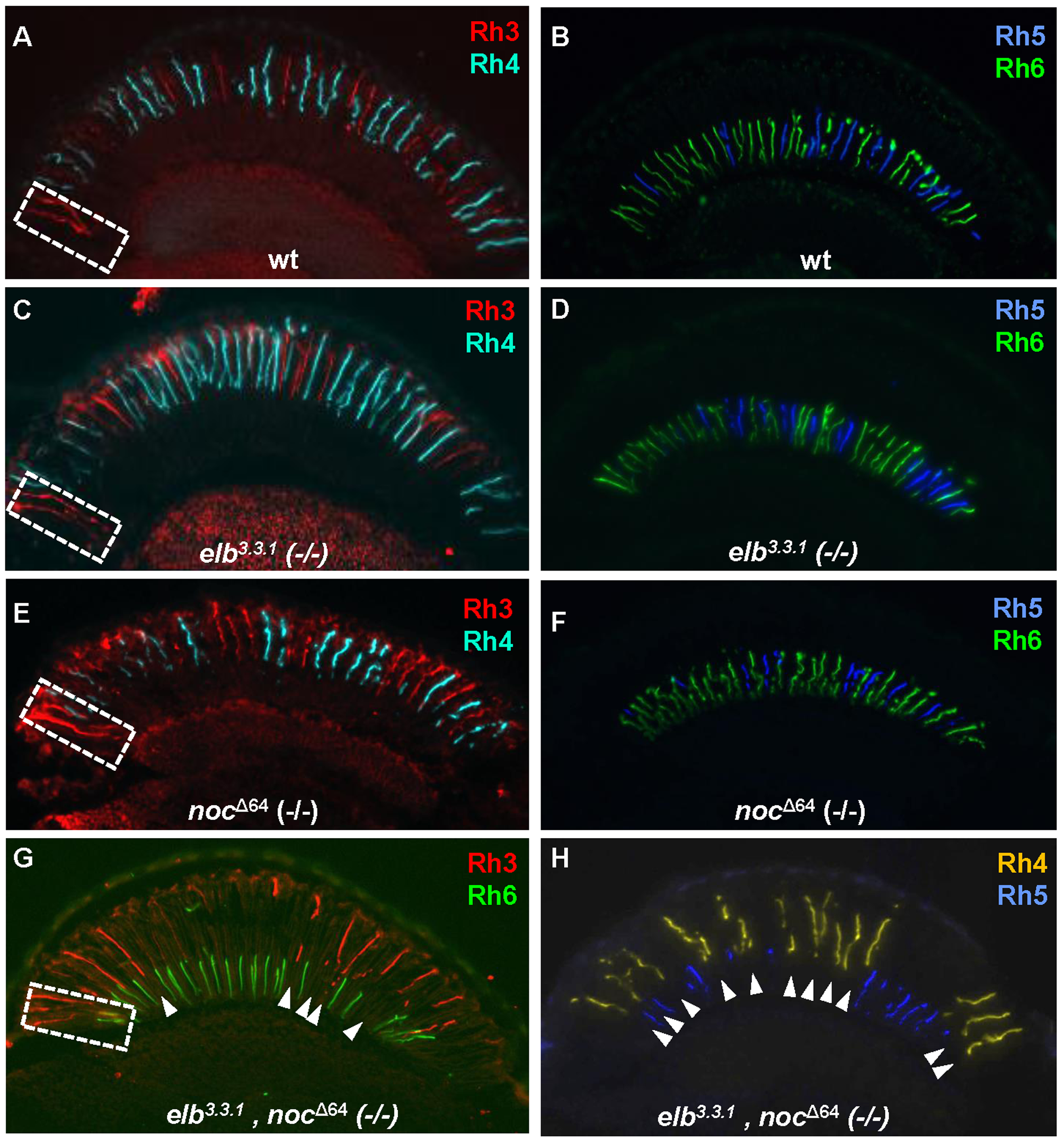

Supplement: Figure S2 — elb, noc single, or double mutants have no phenotype outside the DRA. A,B. Central photoreceptor opsin expression in the wild type retina: R7 opsins Rh3 (red) and Rh4 (cyan) (A), and R8 opsins Rh5 (blue) and Rh6 (green) labeled on Cryostat cross section (B). C,D. Opsin expression in R7 and R8 cells is normal in elb3.3.1 (-/-) single mutants: R7 and R8 in the DRA (dashed white box) are labeled with Rh3 (red), while Rh4 (cyan) in yR7 cells outside the DRA is normal (C). p/y opsin ratios appear normal in both R7 and R8 (D). E,F. No change in opsin expression is visible in homozygous nocΔ64 (-/-) mutant flies. G,H. Coupling of central photoreceptor opsins is normal in elb3.3.1,noc Δ64 (-/-) double mutants: coupled expression of Rh3 (red) and Rh6 (green) within the same ommatidium is not observed. The same is true for coupling of Rh4 (yellow) and Rh5 (blue). Positively labeled R7 cells are always coupled with gaps in the R8 opsin pattern, as reported for wild type flies. (TIF) [file pgen.1004210.s002.tif]

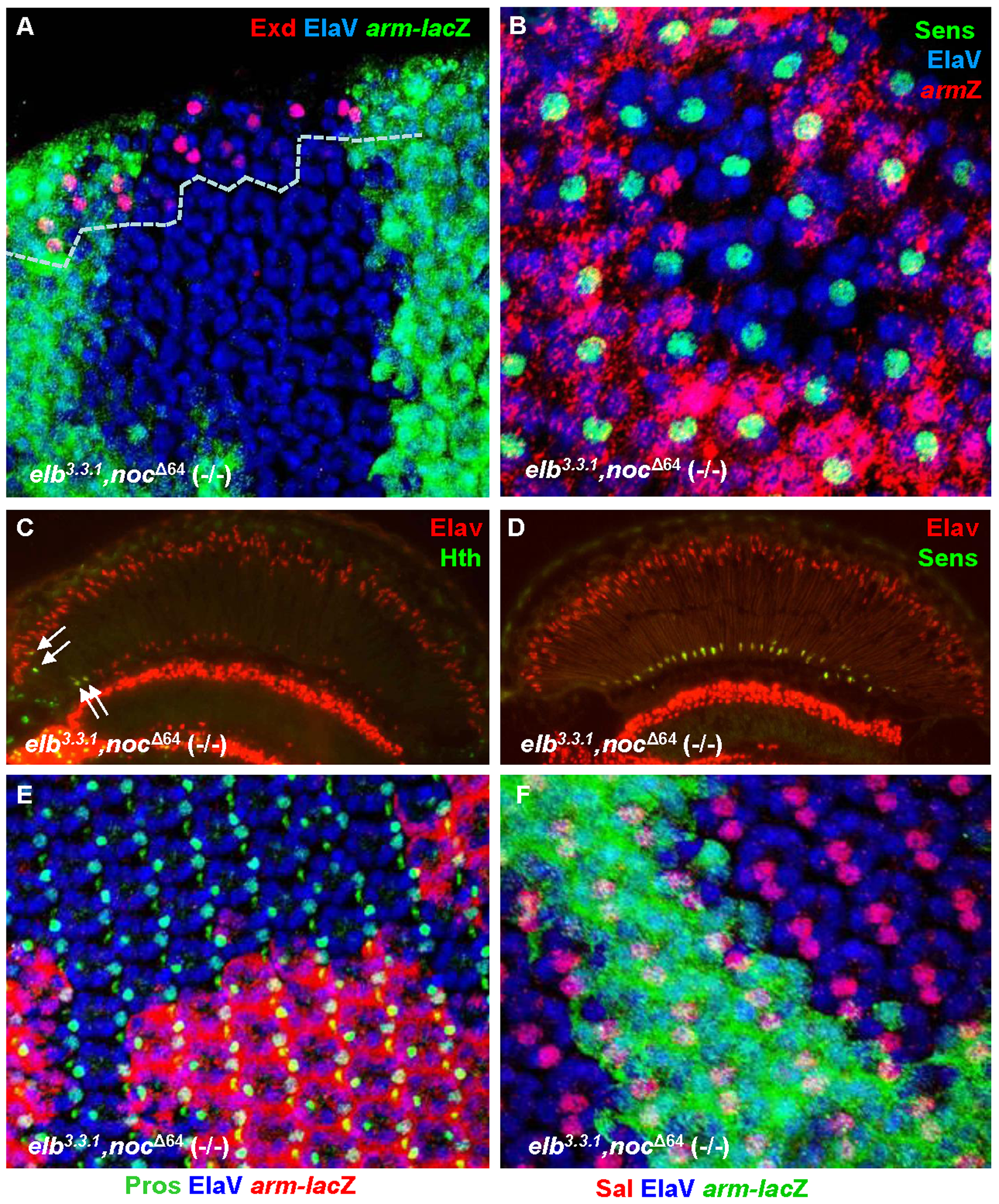

Supplement: Figure S3 — Photoreceptor specification is normal in elb,noc double mutants. A. During pupation, DRA ommatidia labeled with Exd (red) are specified normally in elb3.3.1,noc Δ64 (-/-) double mutant clones (marked by the absence of arm-lacZ, green). B. Similarly, R8 cells are specified correctly, as seen with Sens (green) being unaltered in double mutant clones (marked by the absence of arm-lacZ, red). C,D. Both situations remain indistinguishable from wild type flies throughout adulthood. E. Specification of inner photoreceptors R7 is normal in elb3.3.1,noc Δ64 double mutant clones; labeled: Pros (green) and arm-lacZ (red). F. Expression of inner photoreceptor marker Spalt (red) is unaltered inside homozygous elb.noc clones. (TIF) [file pgen.1004210.s003.tif]

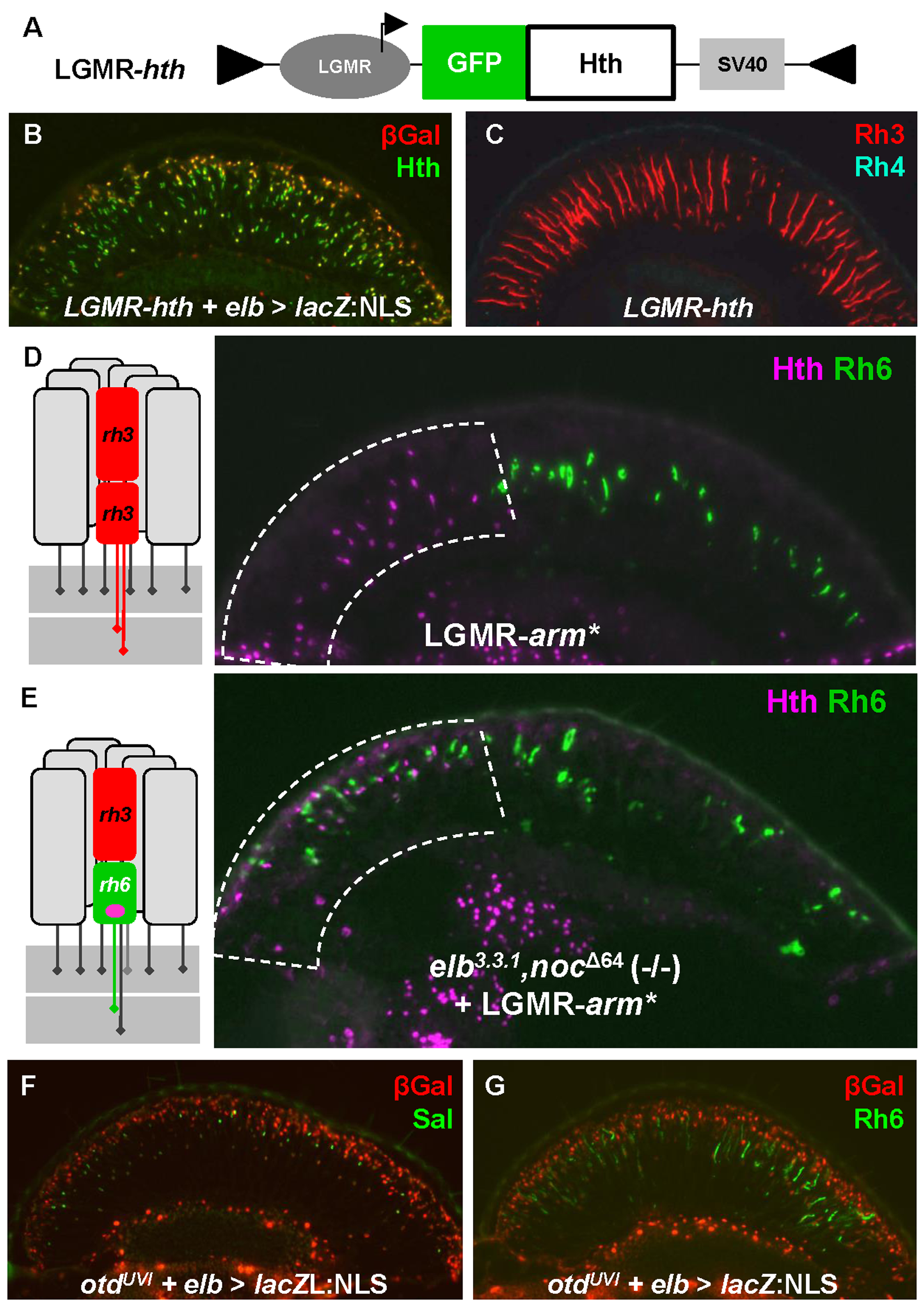

Supplement: Figure S4 — elb, noc, and hth expression in different genetic backgrounds. A. Schematic of LGMR-GFP:Hth transgenes generated for this study. B. In the adult eye, Hth (green) is over-expressed in all photoreceptors, by the direct fusion transgene LGMR:GFP:Hth, and expression of elb-GAL4 is not expanded (visualized using UAS-lacZ:NLS, red). C. The entire eye is transformed into DRA ommatidia, as previously shown using the GAL4/UAS technique: Rh3 (red) is expanded throughout the retina. Rh4, Rh5, and Rh6 are lost (not shown). D,E. Ectopic activation of the wingless pathway (LGMR-arm*) leads to an expansion of Rh3/Rh3 coupled DRA ommatidia (schematic, left) across the dorsal half of the retina (dashed white box), with Rh6 and Hth expression excluding each other. E. Co-expression of Hth and Rh6 in the dorsal eye in elb,noc double mutants, when the Wingless pathway is ectopically activated using LGMR-arm*, resulting in ‘odd-coupled’ Rh3/Rh6 ommatidia (schematic, left). F,G. Expression of elb-GAL4 (red) is unchanged in eye-specific mutants loss-of-function mutants of otd (ocelliless, oc), called otdUVI. Note expansion of Rh6 expression (green) into outer photoreceptors, in these mutants (G), with Rh6-positive rhabdomeres spanning the entire thickness of the retina. (TIF) [file pgen.1004210.s004.tif]

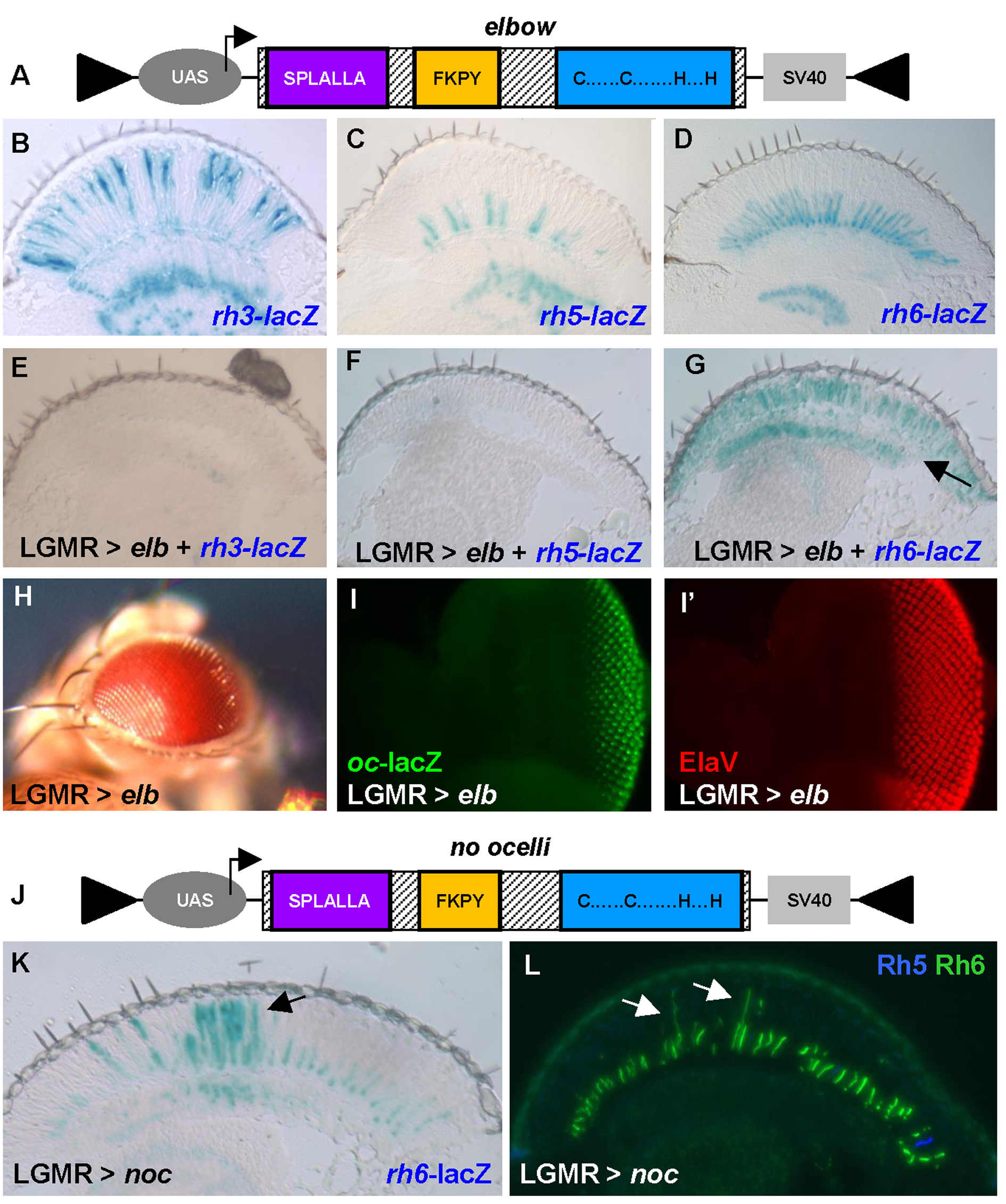

Supplement: Figure S5 — Gain-of-function phenotypes of elbow and no ocelli. A. Schematic showing structure of UAS-elb transgenes used for mis-expression of wild type Elbow protein. B–G. The gain-of-function opsin phenotype obtained with UAS-elb phenocopies otdUVI mutants [49]. Wild type expression (X-Gal staining on Cryostat cross-sections) of rh1-lacZ (B), rh3-lacZ (C), and rh6-lacZ (D). Expression of rh3- and rh5-lacZ was completely lost in LGMR > elb flies (E, F). Expression of rh6-lacZ was expanded into outer photoreceptors (G), as seen by labeled projections into the lamina (black arrow). Expression of rh1- and rh4-lacZ was normal (not shown). H. Eye phenotype obtained when over-expressing elbow in all photoreceptors, using LGMR-GAL4: the compound eye gets shiny and slightly rough. I. Over-expression of wild type Elb protein does not repress transcription of oceliless (Otd). Expression of oc-lacZ (green) is not affected in eye imaginal discs of LGMR > elb flies, double-labeled with Anti-ElaV (red, I′). J. Schematic of UAS-noc transgenes used. K,L. The no ocelli gain-of-function opsin phenotype is weak: While all other opsins are expressed normally, only rh6 shows a weak expansion into outer photoreceptors, as seen with rh6-lacZ (K; black arrow) and Anti-Rh6 signals (green) (L, white arrows) spanning the entire thickness of the adult retina. (TIF) [file pgen.1004210.s005.tif]

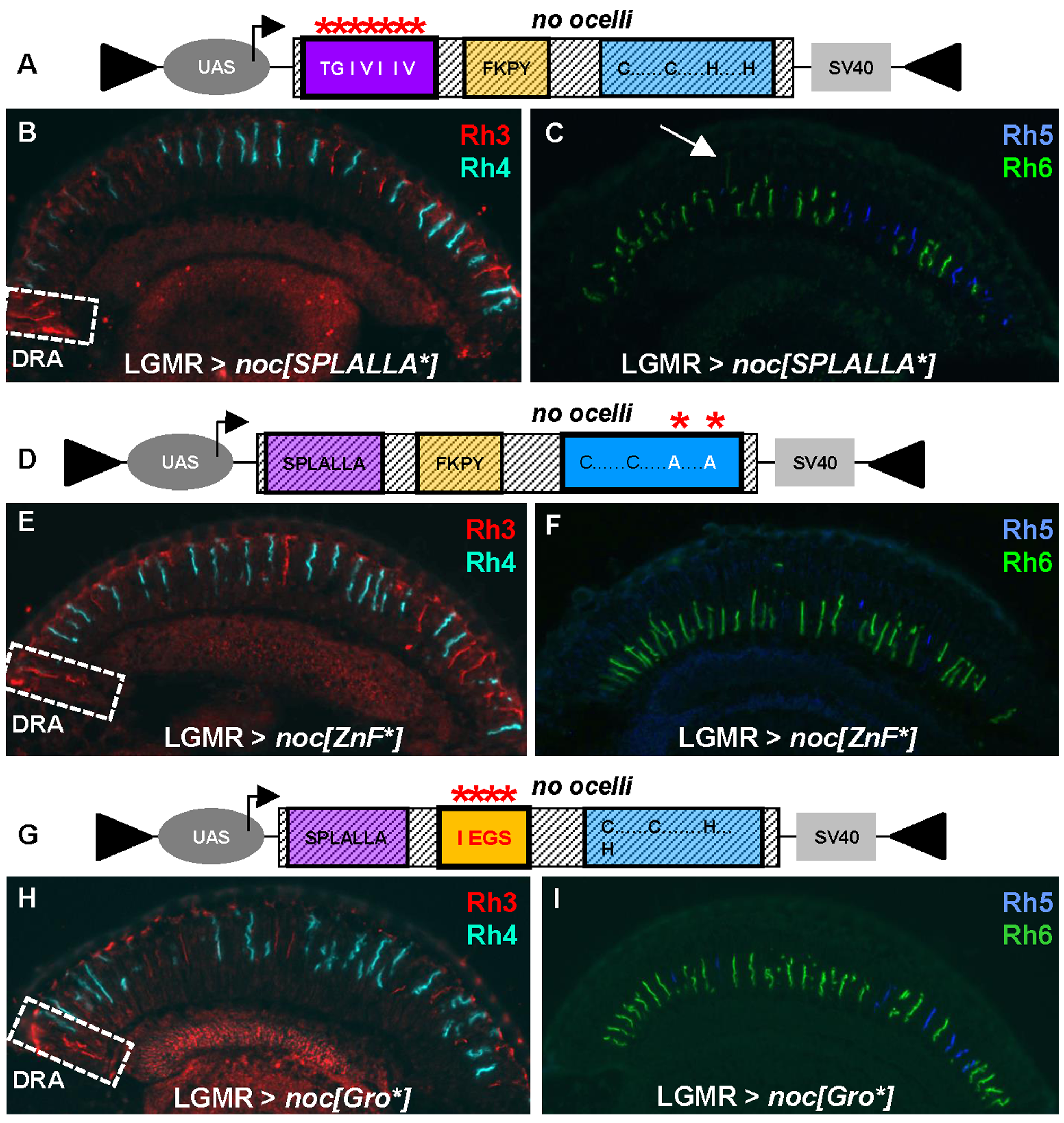

Supplement: Figure S6 — Over-expression of mutated forms of No ocelli. A. Schematic of UAS-transgene generated for mis-expression of Noc protein with a mutated Sp/SPLALLA motif (see Materials and Methods). B. DRA specification and R7 opsin expression is not affected by ectopic Noc[SPLALLA*]. C. R8 opsin expression is mildly affected: Rh6 expression (green) is expanded into some outer photoreceptors (white arrow), while Rh5 expression (blue) is normal. D. Schematic of UAS-transgene generated for mis-expression of No ocelli protein with a mutated Groucho-binding motif (FKPY → IEGS; see Materials and Methods). E+F. DRA specification and inner photoreceptor expression are not affected by ectopic over-expression of Noc[Gro*]. G. Schematic of UAS-transgene generated for mis-expression of Noc protein with a mutated zinc finger, where both zinc finger Histamines have been mutated to Alanines (see Materials and Methods). H, I. DRA specification and inner photoreceptor expression are not affected by ectopic over-expression of Noc[ZnF*]. (TIF) [file pgen.1004210.s006.tif]

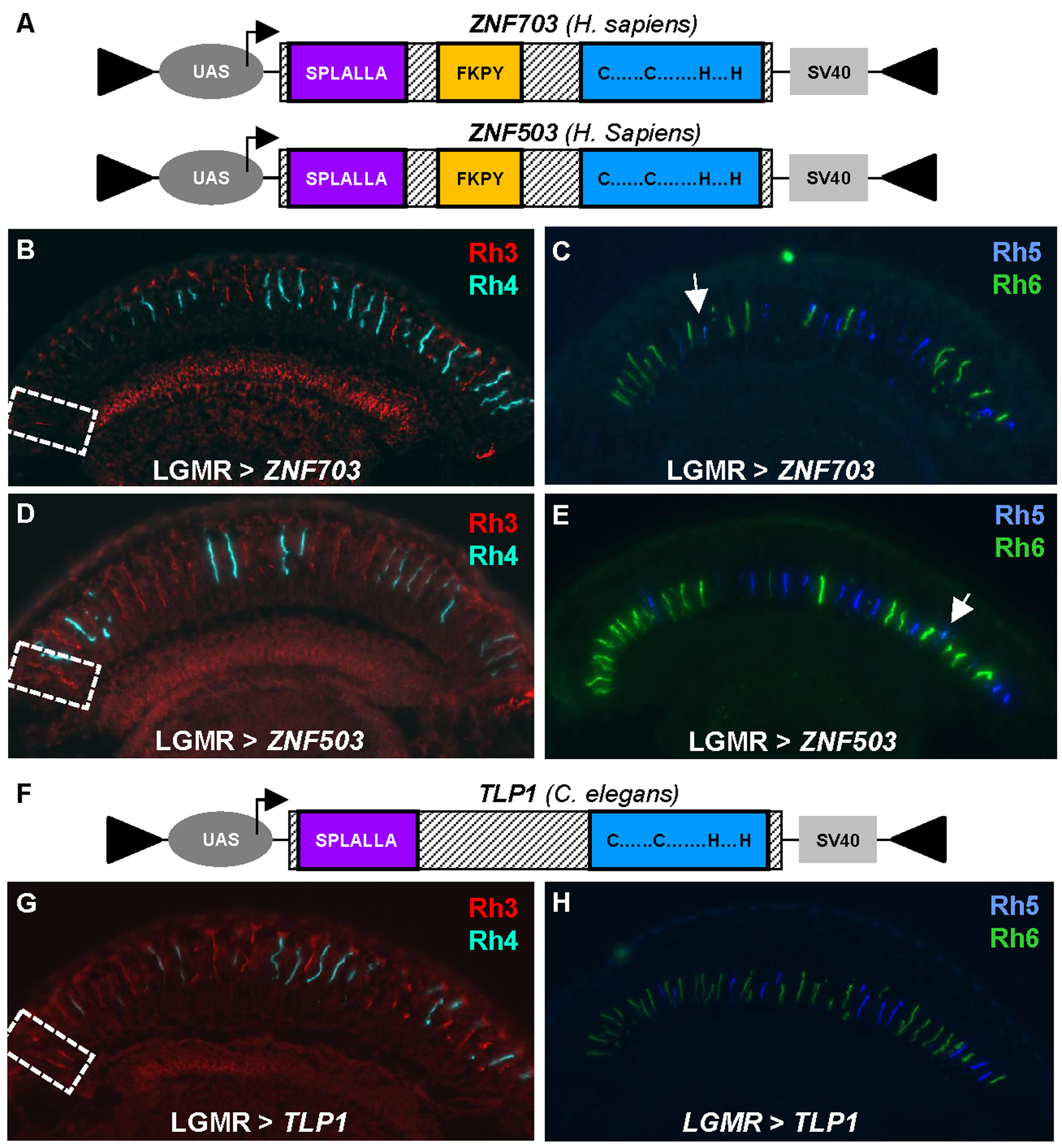

Supplement: Figure S7 — Over-expression of worm and human homologues of Elb/Noc. A. Schematic of UAS-transgene generated for mis-expression of the human homologues of Elb/Noc: ZNF703 and ZNF503. B,D. DRA specification and R7 opsin expression are not affected by ectopic over-expression of ZNF703 (B), or ZNF503 (D). C,E. R8 opsin expression is mildly affected by ectopic over-expression of ZNF703 (C), or ZNF503 (E): occasional co-expression of Rh5 (blue) and Rh6 (green) is observed (white arrows), for both homologues. F. Schematic of UAS-transgene generated for mis-expression of the C. elegans homologue TLP1. G,H. DRA specification and inner photoreceptor expression is not affected by ectopic over-expression of UAS-TLP1. (TIF) [file pgen.1004210.s007.tif]

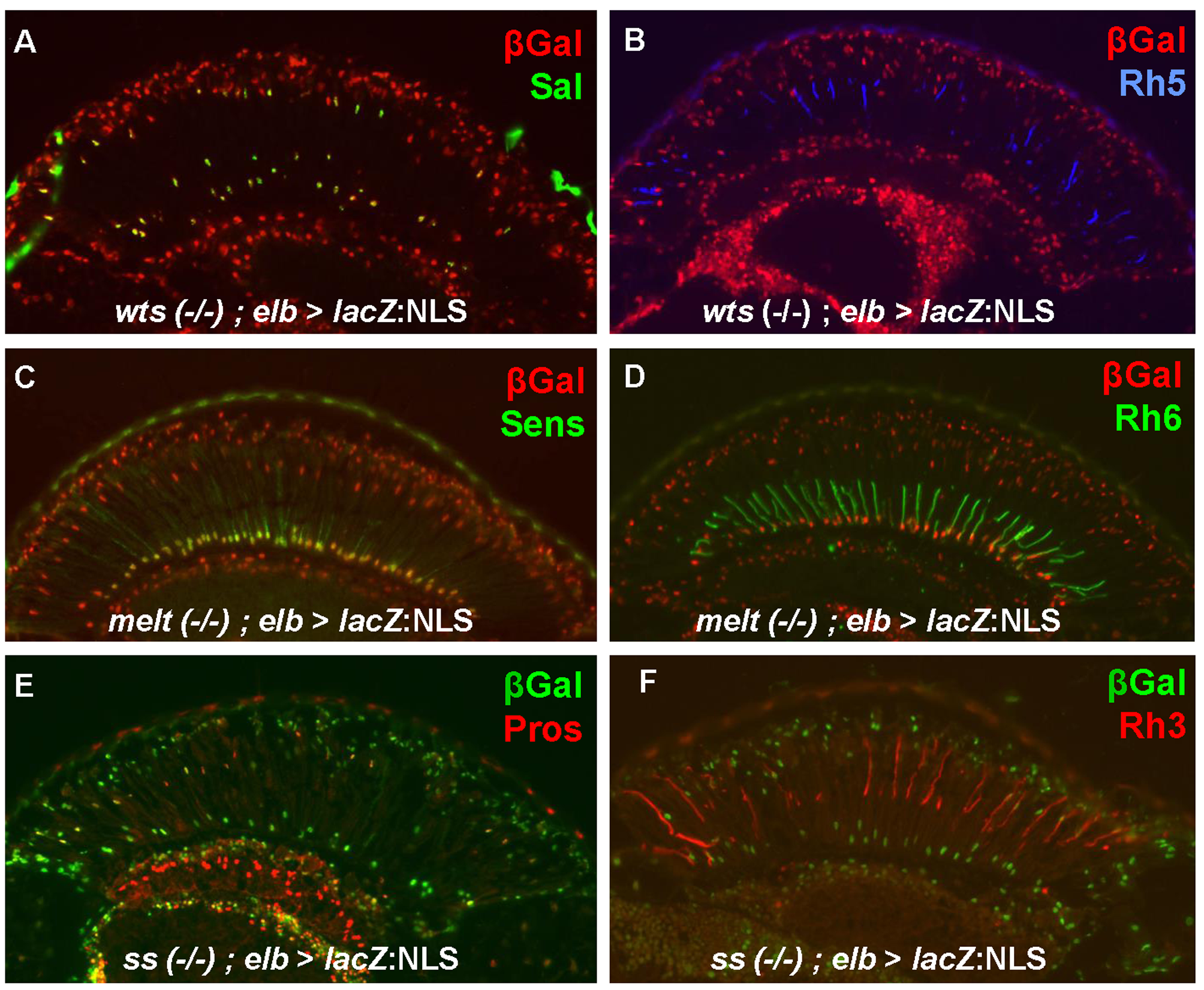

Supplement: Figure S8 — elb, noc expression is normal in warts, melted, and spineless mutants. A,B. Expression of elb-GAL4 (red) is not affected in homozygous mutants for warts (Dlats, wts). Note how expression of pR8 opsin Rh5 (blue) is expanded in these mutants. C,D. Expression of elb-GAL4 (red) is not altered in homozygous mutants for melted (melt). Note how expression of yR8 opsin Rh6 (green) is expanded in these mutants. E,F. Expression of elb-GAL4 (green) is not altered in homozygous spineless (ss) mutants. Note how expression of pR7 opsin Rh3 (red) is expanded in these mutants, while yR7 opsin Rh4 is lost. (TIF) [file pgen.1004210.s008.tif]
